# Critical components of ‘Early Intervention in Psychosis’: a national retrospective cohort study

**DOI:** 10.1192/bjp.2025.126

**Published:** 2025-06-26

**Authors:** Ryan Williams, Ed Penington, Veenu Gupta, Alan Quirk, Apostolos Tsiachristas, Michelle Rickett, Carolyn A Chew-Graham, David Shiers, Paul French, Belinda Lennox, Alex Bottle, Mike J Crawford

**Affiliations:** 1 Division of Psychiatry, Imperial College London, London, UK; 2 Royal College of Psychiatrists, London, UK; 3 Department of Psychiatry, University of Oxford, Oxford, UK; 4 Department of Psychology, Durham University, Durham, UK; 5 School of Medicine, Faculty of Medicine and Health Sciences, Keele University, Newcastle, UK; 6 Department of Research and Innovation, Pennine Care NHS Foundation Trust, Lancashire, UK; 7 School of Public Health, Imperial College London, London, UK

**Keywords:** early intervention, psychosis, schizophrenia, care coordination, caseload, clozapine

## Abstract

**Background:**

Psychotic disorders are severe mental health conditions frequently associated with long-term disability, reduced quality of life, and premature mortality. Early Intervention in Psychosis (EIP) services aim to provide timely, comprehensive packages of care for people with psychotic disorders. However, it is not clear which components of EIP services contribute most to the improved outcomes they achieve.

**Aims:**

We aimed to identify associations between specific components of EIP care and clinically significant outcomes for individuals treated for early psychosis in England.

**Method:**

This national retrospective cohort study of 14874 EIP service users examined associations between twelve components of EIP care and outcomes over a three-year follow-up period, by linking data from the National Clinical Audit of Psychosis (NCAP) to routine health outcome data held by NHS England. The primary outcome was time to relapse, defined as psychiatric inpatient admission or referral to a crisis resolution (home treatment) team. Secondary outcomes included duration of admissions, detention under the Mental Health Act, emergency department and general hospital attendances, and mortality. We conducted multilevel regression analyses incorporating demographic and service-level covariates.

**Results:**

Smaller care coordinator caseloads and the use of clozapine for eligible service users were associated with reduced relapse risk. Physical health interventions were associated with reductions in mortality risk. Other components, such as cognitive behavioural therapy for psychosis (CBTp), showed associations with improvements in secondary outcomes.

**Conclusion:**

Smaller caseloads should be prioritised and protected in EIP service design and delivery. Initiatives to improve the uptake of clozapine should be integrated into EIP care. Other components, such as CBTp and physical health interventions, may have specific benefits for eligible service users. These findings highlight impactful components of care and should guide resource allocation to optimise EIP service delivery.

**Funding:**

National Institute for Health and Care Research (NIHR), UK.

## Introduction

Psychotic disorders affect approximately 1% of the global population and profoundly impact health and quality of life.^1, 2^ Prognoses following a first episode of psychosis (FEP) vary, but many people experience recurrent relapses, long-term disability, social exclusion, and high levels of mental health service use.^3^ People with psychosis are also at increased risk of poor physical health, with mortality rates several times higher than the general population.^4, 5^ Evidence indicates that intensive treatment during the early stages of psychosis can mitigate long-term impacts.^6–8^ Consequently, Early Intervention in Psychosis (EIP) services have been established as a key element of mental health care in the United Kingdom (UK)^9^ and internationally.^10^ EIP services provide a comprehensive package of evidence-based treatments, emphasising prompt access, multidisciplinary care, and recovery-oriented approaches. EIP involvement has been shown to improve outcomes compared with treatment as usual,^3, 11^ in a cost-effective manner.^12^ However, despite calls for research into the ‘active ingredients’ of EIP care, it remains unclear which specific components drive these improved outcomes.^13^ A recent component meta-analysis suggested that care coordination and psychological interventions are key, but findings were limited by reliance on symptom-based outcomes and an inability to isolate the impact of specific components.^14^ Exact service models differ internationally,^15^ and in the UK the National Clinical Audit of Psychosis (NCAP) identified significant variation in care delivery between services^16^ in spite of implementation guidelines.^17^ The impact of this variation on outcomes remains unknown.

### Aims

This retrospective cohort study of 14874 individuals aimed to identify associations between EIP care components and real-world outcomes such as relapse, compulsory hospitalisation, and mortality, using linked data from the NCAP and routine health records. By pinpointing components linked to positive outcomes, we aimed to provide actionable insights to enhance EIP delivery, inform policy and resource allocation across mental health services and ultimately better support individuals experiencing psychosis.

## Method

This study follows the STROBE guidelines for observational studies.^18^ A full checklist and deviations from the published protocol^19^ are provided in the appendix. This study was informed by service users and carers and their priorities for research.

### Study Design

We conducted a retrospective cohort study using data from the NCAP and routine health datasets, over a three-year period. The NCAP is a quality improvement programme led by the Royal College of Psychiatrists which provides high-quality data on psychosis care.^16^ Since 2017, the NCAP has recorded patient-level data from every EIP service in England.

Our cohort comprised 14,874 individuals treated by all EIP services in England during 2019–2020. During each NCAP round a random sample of 100 patients were selected from each service. Where a service’s total caseload comprised <100 eligible patients, all were included.

Inclusion criteria were:

- Diagnosed ‘first episode’ of any ‘non-organic’ psychotic disorder. To facilitate inclusivity and real-world generalisability of results, no specific diagnostic framework such as ICD or DSM was specified.
- Under EIP care for >6 months at NCAP audit date (April 2019 or 2020).
- Aged 14-65 – reflecting EIP access standards.^17^

Patients were excluded from the NCAP (and therefore this study) if they had a diagnosis of psychosis due to an ‘organic cause’.

NCAP exposure data (care components delivered by EIP services) were linked to routine health outcome data for a three-year follow-up period ending January 2023. Linked datasets were the Mental Health Services Data Set (MHSDS), Hospital Episode Statistics (HES), the Emergency Care Data Set (ECDS) and the ONS Civil Registration Death dataset. Data were pseudonymised and stored within the Office for National Statistics Secure Research Service (SRS).

### Exposure Variables

Our exposures are specific components of care specified by the National Institute for Health and Care Excellence (NICE) as necessary constituents of EIP:^20^ receipt of an antipsychotic, receipt of ‘cognitive behavioural therapy for psychosis’ (CBTp), receipt of a family intervention, receipt of vocational support, receipt of a carer-focused intervention, offer and initiation of clozapine where appropriate (patients were eligible if they had ‘treatment-resistant’ symptoms i.e. inadequate response to two previous antipsychotics), receipt of NICE-approved EIP physical health interventions (smoking/ alcohol/ psychoactive substance cessation, weight reduction), average care coordinator caseload size per service, and waiting time (whether waiting time standard was met prior to initiation of EIP treatment).

### Outcome Variables

Our primary outcome is time to ‘relapse’ indicated by inpatient admission or referral to a ‘crisis resolution and home treatment team’ (CRHTT). Secondary outcomes are time to inpatient admission or CRHTT referral alone, duration (bed days) of inpatient stay over the follow-up period, time to detention under the Mental Health Act, number of acute general hospital admissions and Emergency Department attendances, and all-cause mortality.

### Statistical Analyses

Analyses were performed using ‘R’.^21^ Initially we generated descriptive statistics for all exposure variables, outcome measures and covariates, and used unadjusted tests to explore pairwise associations.

Next we conducted multivariable analyses using Cox regression for time-to-event outcomes, and zero-inflated negative binomial regression for count outcomes. For time-to-event outcomes, the time of exposure was taken as date of the relevant NCAP audit period for each individual (1st April 2019 or 1st April 2020). Multilevel models were used to account for clustering effects within EIP services and were adjusted for covariates identified via a Directed Acyclic Graph (DAG), including age, sex, ethnicity, employment status and prior admissions (within the data extract window, i.e. post-April 2016) - see ‘Detailed Statistical Methods’ and Figure A1 in the appendix for further details. Interactions (between all demographics variables and exposures) were checked for each outcome and included in model exploration if influential.

Final models were selected using a ‘goodness-of-fit’ approach, aiming to establish models that captured as much information in the data with as few parameters as possible (see appendix for further details of statistical methods). We assessed model assumptions using appropriate statistical tests.

Given the large number of comparisons, we adopted a significance threshold of p≤0.001 to reduce spurious findings. This threshold was chosen as a heuristic to focus on robust associations rather than applying formal corrections, which may be overly conservative in observational analyses. Results with p-values between 0.001 and 0.05 were considered suggestive but not definitive. This threshold of p ≤ 0.001 is broadly consistent with the significance levels used when controlling the type 1 error rate at 5% in large datasets.^22^ Missing data for service-level variables were imputed using multiple imputation, with sensitivity analyses to test robustness (see appendix).

## Results

Data were obtained for 14,874 participants. Mean age was 33.5 years (SD 11.5), and 9225 were male (62.0%). Demographic characteristics and a summary of exposure and outcome variables are given in Table 1, with more detailed breakdowns (specific ethnicity categories, distribution of comorbidities) available in Figures A2-4, and Tables A1-3 in the appendix.

### Primary Outcome

Associations between exposure variables, covariates and adjusted hazard rates of relapse (i.e. the risk of a psychiatric admission or referral to a CRHTT, occurring at any given time point during the follow-up period) are reported in Table 2. Hazard rate ratios indicate the likelihood that a participant with the given exposure experienced the outcome at any given time point, relative to the ‘reference’ category for each variable. HR > 1 indicates an increased likelihood of outcome, while HR < 1 indicates a decreased likelihood.

We found strong evidence that smaller care coordinator caseloads and the use of clozapine were associated with reduced relapse rates. Hazard rates were increased by 2% for each additional person on the caseload of an individual’s care coordinator (HR 1.02, 95% CI 1.01-1.02, p<0.001).

For clozapine, we found substantially increased hazard rates (compared with those who were not eligible to receive clozapine) for those who were eligible to receive it and refused it (HR 1.38, 95% CI 1.17–1.63, p<0.001), or who were not offered it (HR 1.50, 95% CI 1.36–1.67, p<0.001). However, those who were eligible for clozapine and received it showed no significant difference to ineligible individuals (HR 0.97, 95% CI 0.85–1.10, p=0.614).

**Figure 1:**
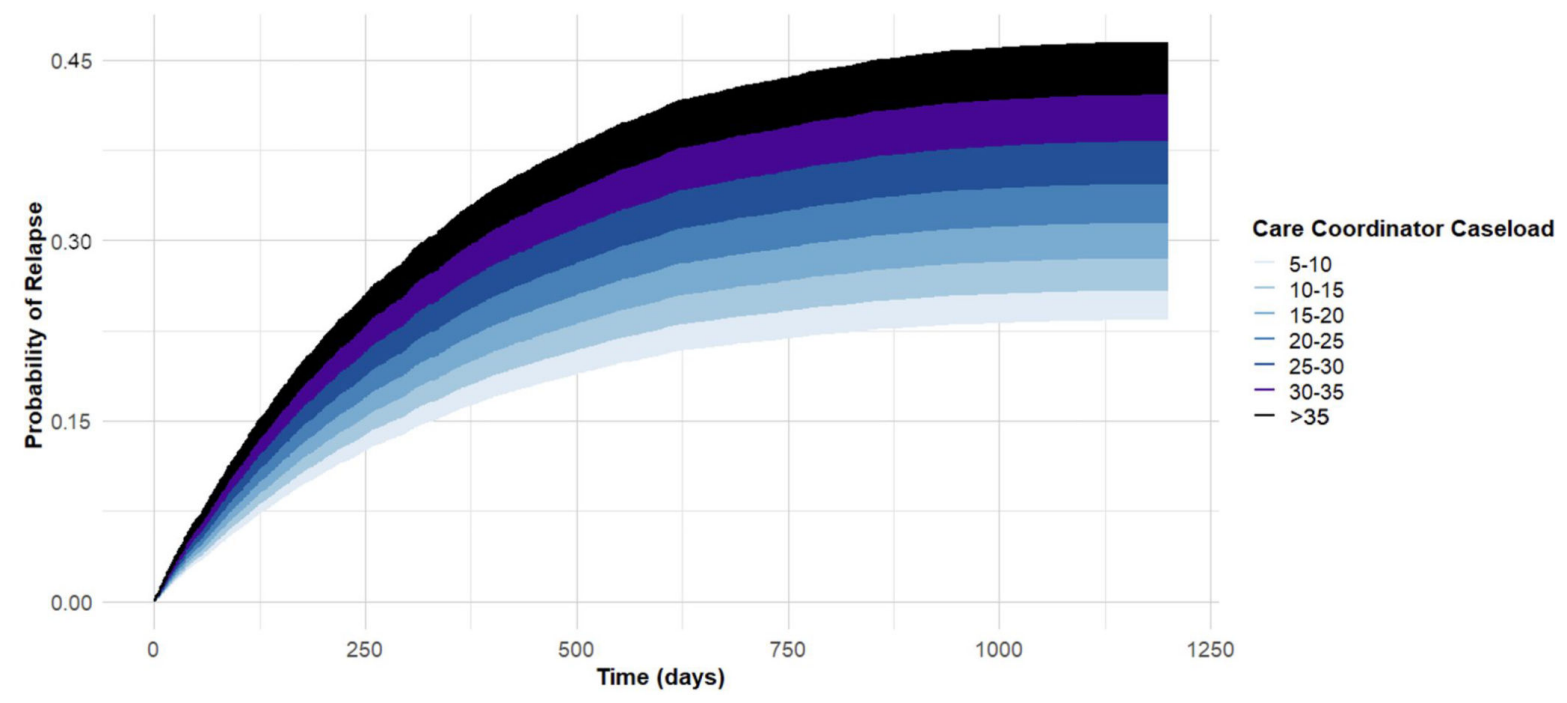
Probability of relapse by average care coordinator caseload This plot illustrates the cumulative probability of relapse over time (in days) when care coordinator caseload is varied for an otherwise ‘typical’ Early Intervention in Psychosis (EIP) patient. This typical patient is defined as having mean age and the most prevalent characteristics across other variables (e.g., Male, White, Unemployed, in receipt of CBTp etc). Survival times were probabilistically simulated using parameterized hazard rates for each group from a Cox proportional hazards model (with relapse probabilities increasing only at discrete event times) reflecting the plausible time-to-relapse patterns from the real population.

**Figure 2:**
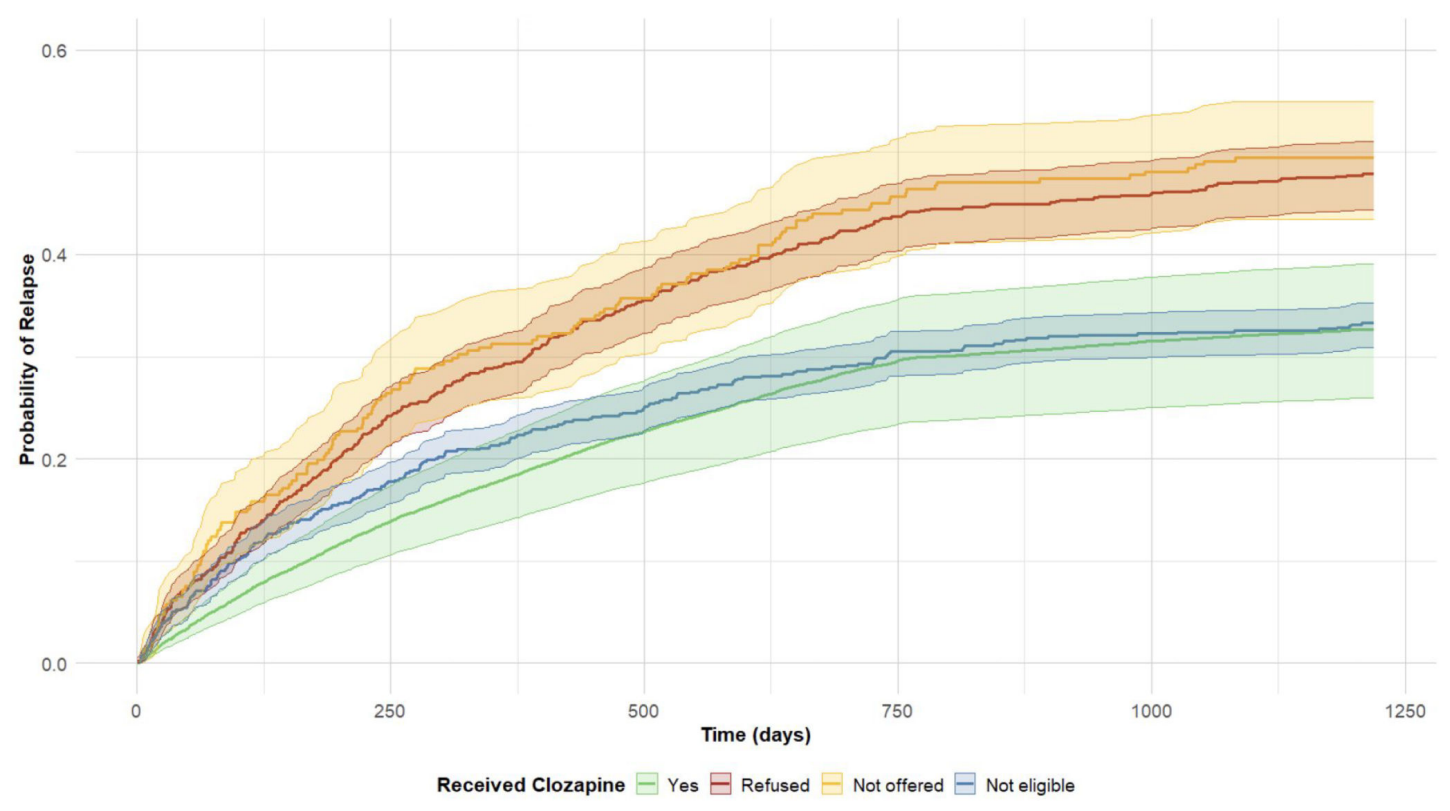
Probability of relapse by eligibility/ receipt of clozapine This plot illustrates the cumulative probability of relapse over time (in days) based on clozapine eligibility and receipt for an otherwise ‘typical’ Early Intervention in Psychosis (EIP) patient. This typical patient is defined as having mean age and the most prevalent characteristics across other variables (e.g., Male, White, Unemployed, in receipt of CBTp etc). Survival times were probabilistically simulated using parameterized hazard rates for each group from a Cox proportional hazards model (with relapse probabilities increasing only at discrete event times) reflecting the plausible time-to-relapse patterns from the real population.

Effects on these variables on probability of relapse over the follow-up period are illustrated in cumulative incidence plots in Figures 1 and 2.

Hazard rates were also higher for those requiring substance use interventions, regardless of whether interventions were refused (HR 1.45, 95% CI 1.32–1.59, p<0.001) or received (HR 1.52, 95% CI 1.42–1.63, p<0.001). No other components of care were associated with significant differences in rates of relapse.

Individuals from Black, Asian and Minority Ethnic (BAME) groups had higher relapse rates than White individuals (HR 1.18, 95% CI 1.11–1.26, p<0.001), and relapse rate decreased by 2% per additional year of age (HR 0.98, 95% CI 0.98–0.98, p<0.001). Individuals who had already had an inpatient admission prior to the NCAP census period had substantially higher hazard rates of subsequent relapse compared with those who had not (HR 2.25, 95% CI 2.11–2.40, p<0.001).

### Secondary Outcomes

Findings for time-to-event secondary outcomes (psychiatric admission, CRHTT referral, Mental Health Act detention, mortality) and count outcomes (hospital admissions, ED attendances, admission duration) are detailed in Tables A4-A10 (appendix).

For the outcomes ‘time to psychiatric hospital admission’, ‘time to CRHTT referral’, and ‘time to detention under Mental Health Act’, findings broadly mirrored those for relapse. This was unsurprising given the high degree of correlation between these outcomes (those with increased likelihood of a relapse overall also had increased likelihood of an inpatient admission or a CRHTT referral by definition but also tended to have increased likelihood of detention under the Mental Health Act). Poorer outcomes were seen for higher care coordinator caseloads, those who were eligible for clozapine and did not receive it (compared with those who were ineligible) and those who required interventions for substance use.

However, there were some specific differences. For CRHTT referral specifically there was suggestive evidence that those who were eligible for clozapine and received it might have lower (rather than equivalent) hazard rates than ineligible individuals (HR 0.85, 95% CI 0.73–0.99, p=0.033). In addition, those who received carer interventions had higher rates of referral than those with no identified carer involved (HR 1.16, 95% CI 1.07–1.25, p<0.001).

For psychiatric admission specifically, there was very weak evidence that those who received CBTp had reduced hazard rates compared with those who were not offered it (HR 0.91, 95% CI 0.83-1.00, p =0.057).

For rates of detention under the Mental Health Act, clozapine recipients had lower (rather than equivalent) hazard rates than those ineligible for clozapine (HR 0.82, 95% CI 0.68–0.98, p<0.001).

Detention rates were also reduced for those that received CBTp (HR 0.85, 95% CI 0.77–0.94, p=0.001), but were higher for BAME (HR 1.47, 95% CI 1.36–1.59, p<0.001) or other non-white ethnic groups (HR 1.27, 95% CI 1.11–1.44, p<0.001) than for white individuals.

For mortality, hazard rates were substantially higher for individuals requiring interventions for alcohol use who either refused them (HR 2.59, 95% CI 1.46-4.58, p<0.001) or did not receive them for some other reason (HR 1.80, 95% CI 1.06–3.39, p<0.001) compared with those who did not require them. However, mortality rates were not increased for those who required an intervention and received it (HR 1.14, 95% CI 0.66–1.97, p=0.638). A similar pattern was seen for weight loss interventions, with increased mortality for those refusing them (HR 1.85, 95% CI 1.05–3.24, p<0.001) or not receiving them for other reasons (HR 2.36, 95% CI 1.32–4.22, p<0.001), but not for those who received them. Mortality rates were increased for those who received interventions for substance use, compared with those who did not require them (HR 2.50, 95% CI 1.74-3.59, p<0.001); and increased by 7% for each one-year increase in age (HR 1.07, 95% CI 1.05-1.08, p<0.001).

For the following count outcomes, incidence rate ratios indicate the relative frequency with which a participant with the given exposure experienced the outcome, relative to the ‘reference’ category for each variable. IR > 1 indicates an increased frequency of outcome, while IR < 1 indicates a decreased frequency.

For the outcome ‘admission duration (bed days)’, we found suggestive evidence that individuals who were eligible for clozapine but did not receive it spent longer admitted over the follow-up period than those who were not eligible – both those who were not offered it (IR 1.47, 95% CI 1.07–2.08, p=0.024) or weaker evidence for those that refused it (IR 1.67, 95 CI 1.00–3.08, p=0.053). Shorter admission durations were observed for those offered CBTp, particularly if they received it (IR 0.73, 95% CI 0.59–0.89, p=0.002), though reduced durations were also seen in those that refused it (IR 0.84, 95% CI 0.66–0.96, p=0.037).

Clozapine recipients had lower Emergency Department attendance rates than those who were ineligible for clozapine (IR 0.75, 95% CI 0.66-0.86, p<0.001). There was suggestive evidence that those who had an identified carer might also have lower rates of ED attendance than those who had not – including those who received a carer intervention (IR 0.90, 95% CI 0.85-0.96, p=0.002) and those who did not (IR 0.90, 95% CI 0.84-0.96, p=0.002). Rates of attendance were increased for those who received interventions for alcohol (IR 1.37, 95% CI 1.23–1.54, p<0.001), smoking (IR 1.18, p5% CI 1.10–1.26, p<0.001), and substance use (IR 1.37, 95% CI 1.26–1.48, p<0.001).

For ‘general hospital admission’, no components of care were associated with improved incidence rates. Increased rates were observed for those who received interventions for smoking (IR 1.22, 95% CI 1.12-1.32, p<0.001), alcohol (IR 1.29, 95% CI 1.13-1.47, p<0.001), or substance use (IR 1.24, 95% CI 1.13-1.36, p<0.001).

### Missing Data and Sensitivity Analyses

Our chosen outcomes are mandatory submissions for NHS England. The absence of data for an outcome was interpreted by NHS England as the patient not having experienced the outcome. There is a possibility that in some cases, missing data was due to incomplete or incorrect recording rather than a true absence of the outcome. If the likelihood of recording was associated with patient characteristics or outcomes, this could introduce a risk of informative censoring. However, due to the nature of the data, it was not possible to test for this.

The data supplied from the NCAP were extremely comprehensive with very few missing values – the only exceptions were the service-level exposure variables (care coordinator caseload per service and proportion meeting waiting time standard per service), which had been compiled from sources other than the case-note audit. For a more detailed breakdown of missing data see ‘Missing Data’ and Tables A5-6 in the appendix.

Our sensitivity analyses examining the effects of different approaches to imputing missing data for service-level exposure variables (care coordinator caseload size and waiting times) resulted in comparable findings (full results are available in the appendix).

## Discussion

This study is the first to examine outcomes from components of EIP care using population-level routine health outcome data in the UK. Our findings suggest that smaller care coordinator caseloads and clozapine use are associated with improved relapse rates, while highlighting potential benefits of other care components including CBTp and physical health interventions. These results offer actionable pathways to optimise EIP services and important principles for supporting people with psychosis across clinical settings.

Smaller care coordinator caseloads were strongly associated with reduced relapse rates, with a 2% increase in hazard per additional patient. Over three years, this equated to a nearly 50% difference in relapse probability between the smallest and largest caseloads. This finding aligns with recent evidence highlighting the importance of quality care coordination in EIP, where it is viewed as a significant advantage compared to other settings such as Community Mental Health Teams.^23^ Smaller caseloads may facilitate more frequent patient contact, but also provide capacity for other activities, such as care coordinator involvement in group-based interventions and liaison with family and carers. These benefits may promote the formation of a stronger therapeutic relationship, as well as allowing for enhanced monitoring and timely intervention. This, in turn, may better enable care coordinators to prevent deterioration, or detect and avert the early signs of relapse.

This finding does contrast with the results of a previous clinical trial – the UK700 study - which concluded that reduced caseload sizes in intensive case management did not confer significant benefits over standard care.^24^ However, UK700 investigated a population with chronic psychosis and prior hospitalisations and was conducted before the widespread implementation of EIP services. This may have diluted the benefits of smaller caseloads, which may be more impactful during the critical early stages of psychosis. Our findings may reflect the greater importance of intensive, personalised treatment in EIP, and we feel they are more valid in the context of modern psychosis care. They align with recent studies identifying care coordination as one of the most impactful elements of an EIP package of care^14^ and associating smaller caseloads with improvements in patient-reported outcomes.^25^

Current standards for EIP implementation in the UK recommend a caseload of 15 per full-time care coordinator^17^ – we would note that the median caseload at services in this study exceeded this (17.4). Published NCAP reports have previously advised that caseloads > 25 are ‘likely to adversely impact on EIP outcomes’ and suggested that directors of operations should ‘work to ensure (caseloads) remain appropriate’,^16^ but this figure was also exceeded for around 15% of our sample. Given that our analysis associated a reduction from a caseload of >25 to 15 with a roughly 25% reduction in relapse probability over three years, we would suggest that the lower end of this range remains a more appropriate target for EIP caseloads, although economic cost-benefit analyses are required to provide concrete recommendations.

Our findings reaffirm clozapine’s established efficacy for the management of psychotic disorders,^26^ including early psychosis.^27^ Although the number of eligible patients was small, we found benefits in reducing rates of relapse, detention under the Mental Health Act, bed days in hospital and emergency department attendance. Eligible individuals not offered or declining clozapine had worse outcomes, while recipients had risks comparable to, or lower than, those ineligible for clozapine – which is striking in light of historically poor outcomes for people with treatment-resistant psychosis.^28^ Treatment resistance is common even in the early stages of psychosis and may be under-identified.^29^ We note that the majority even of those patients identified as eligible for clozapine (i.e. treatment resistant) in this cohort did not receive it (987/1842, 54%). For some of these, clozapine may have been unsuitable for other reasons not evident from our data (e.g. contraindications related to physical health). However, data from the latest round of the NCAP and regional studies show large ongoing variations in the proportion of eligible patients who receive clozapine treated by different service providers.^16, 30, 31^ Addressing underuse of clozapine through clinician training, enhanced monitoring infrastructure, and patient education campaigns remains a critical priority, particularly in EIP.^32–34^

There were few deaths in the cohort, but the association between targeted physical health interventions and reduced mortality emphasises the importance of integrating these (including addiction services) into EIP infrastructure. Those receiving interventions for reducing alcohol use or promoting weight loss had mortality rates comparable to those not requiring them, while refusal or non-offer of interventions was associated with higher mortality risks. These findings highlight the potential for tailored physical health interventions to address the disproportionate physical health burden faced by individuals with psychosis. People who received these interventions did have increased rates of Emergency Department attendance, but this may reflect improved knowledge about accessing healthcare services rather than poorer health outcomes.

There was weak evidence that CBTp was linked to reductions in hospital admissions and bed days, although the influence of ‘confounding by indication’ should be considered (with evidence that those who refused CBTp also had shorter admissions than those who were not offered it, indicating CBTp may be offered disproportionately to those likely to have shorter admissions, whether they then accept or refuse it). It was not significantly associated with relapse overall, and the relatively small effect sizes are consistent with previous studies.^35^ However, there was somewhat stronger evidence that the receipt of CBTp was associated with reduced hazard rates for detention under the Mental Health Act (compared to both those who refused or otherwise did not receive it). In the context of the more modest improvement in overall admission rates, this suggests that those who received CBTp may have a greater proportion of voluntary admissions, possibly due to improved collaboration and an increased likelihood to understand and accept the need for inpatient care without detention.

The lack of clear improvements with other NICE-recommended interventions, such as family interventions and vocational support, is consistent with a recent meta-analysis of components of EIP care.^14^ While these components have demonstrated efficacy in focussed studies,^36–38^ evidence of additional benefit when combined with other EIP components is limited. This may reflect variability in implementation quality, and further research is needed to optimise their delivery in routine clinical practice and bridge the gap to consistent effectiveness.

Finally we would highlight that even controlling for the components received, people from non-white ethnic groups had increased hazard rates of relapse, and higher still rates of detention under the Mental Health Act. This finding unfortunately underscores ongoing systemic inequalities in mental health care,^39, 40^ highlighting the need for targeted outreach, culturally tailored interventions, and initiatives to address racial disparities in EIP and mental health services generally. Continued measurement of demographic-specific outcomes is needed to ensure these measures are effective.

### Strengths and Limitations

We used a large, nationally representative cohort and conducted robust statistical modelling of real-world outcomes using high-quality datasets. Data quality was assured through the NCAP’s rigorous validation processes. However, the study does have some important limitations. The definition of relapse for our primary outcome relied on health service use rather than symptoms or self-defined recovery. As an observational study, residual confounding remains a possibility. For example, those services able to maintain lower caseloads may also have other advantageous characteristics (e.g. resources, staff continuity), which influence outcomes. Associations may also have been influenced by other unmeasured variables such as overall symptom severity, duration of untreated psychosis, or the use of other medications besides antipsychotics, if these differed between groups (i.e. between those who received vs. refused clozapine). This is particularly evident in some results - while antipsychotic medication use overall was associated with increased relapse hazards, this likely reflects the fact that those who do not receive antipsychotic medication (a small proportion of EIP patients) have less severe baseline symptoms, rather than an effect of treatment itself.

Time-to-event variables were measured from the NCAP audit date rather than exact exposure times, introducing potential variability. For instance, some individuals may have completed CBTp before the NCAP, while others may have still been receiving it at the time of the audit period and continued afterward. However, given the large size of our cohort, these variations are likely to attenuate through random distribution across the sample, and we would not expect them to differ systematically between exposure variables. Our categories for demographic variables may not capture more specific differences for groups within these categories (e.g. specific ethnicities or people who were unemployed for differing reasons). Finally, generalisability may be limited to contexts with similar policy and funding frameworks to England.

### Conclusions

Smaller caseloads and increased use of clozapine and physical health interventions should be prioritised in EIP service design and delivery. Policymakers and commissioners should consider adopting caseload size as a quality metric supported by adequate funding, particularly in high-demand areas. Initiatives to improve clozapine uptake and engagement with physical health interventions among eligible individuals could further enhance outcomes. These could include training programs for clinicians, improved infrastructure for monitoring, and education campaigns for patients and carers. Closer collaboration with primary care may also be particularly helpful for improving the delivery of interventions for physical health. Future research should examine optimal caseload thresholds and explore mechanisms underlying the associations we have identified. For instance, qualitative research could explore perspectives of care coordinators and service-users on how lower caseloads might impact the quality and frequency of therapeutic interactions. Real-world testing of optimal EIP caseload thresholds through randomised or quasi-experimental studies, and cost-benefit analyses balancing the costs of increased staffing against improved outcomes, would provide concrete evidence to guide policy and service design. Further research exploring the possible link between CBTp and reductions in compulsory treatment would also be beneficial. Our findings offer a foundation for developing more effective and equitable EIP models, ultimately improving outcomes for individuals with psychosis.

## Supplementary Material

Supplementary Material

## Figures and Tables

**Table 1:** Cohort demographics

DEMOGRAPHIC VARIABLES	
Age	
Mean (SD)	33.5 (11.5)
Median (IQR)	30 (25 – 40)
Gender (%)	
Male	9225 (62.0)
Female (or other)	5649 (38.0)
Ethnicity (%)	
White	9627 (64.7)
Black, Asian and Minority Ethnic Groups	3973 (26.7)
Other/ not recorded	1274 (8.6)
In employment or education (%)	
No	8953 (60.2)
Yes	5921 (39.8)
EXPOSURE VARIABLES	
SERVICE LEVEL	
CARE COORDINATOR CASELOAD	
Mean (SD)	18.5 (5.7)
Min-Max	7.0 – 54.5
Median (IQR)	17.4 (15.1 – 20.4)
PROPORTION WAITING TIME STANDARD MET	
Mean (SD)	74.7 (14.4)
Min-Max	30 - 100
Median (IQR)	75.0 (64.0 – 86.0)
PATIENT LEVEL	
RECEIVED ANTIPSYCHOTIC (%)	
No	924 (6.2)
Yes	13950 (93.8)
RECEIVED CLOZAPINE (%)	
Not eligible	12964 (87.2)
Not offered	987 (6.7)
Refused	291 (1.9)
Yes	630 (4.2)
RECEIVED CBTp (%)	
No	3646 (24.5)
Refused	3975 (26.7)
Yes	7253 (48.8)
RECEIVED FAMILY INTERVENTION (%)	
No	6033 (40.6)
Refused	5508 (37.0)
Yes	3333 (22.4)
RECEIVED CARER INTERVENTION (%)	
Not eligible	4021 (27.0)
No	4724 (31.8)
Yes	6129 (41.2)
RECEIVED EMPLOYMENT INTERVENTION (%)	
No	5838 (39.2)
Refused	4160 (28.0)
Yes	4876 (32.8)
RECEIVED SMOKING INTERVENTION (%)	
Not required	7278 (48.9)
No	1512 (10.2)
Refused	2386 (16.0)
Yes	3698 (24.9)
RECEIVED WEIGHT INTERVENTION (%)	
Not required	8730 (58.7)
No	487 (3.3)
Refused	465 (3.1)
Yes	5192 (34.9)
RECEIVED ALCOHOL INTERVENTION (%)	
Not required	11682 (78.5)
No	1377 (9.3)
Refused	975 (6.6)
Yes	840 (5.6)
RECEIVED SUBSTANCE INTERVENTION (%)	
Not required	10005 (67.3)
No	1243 (8.4)
Refused	1327 (8.9)
Yes	2299 (15.4)
OUTCOME VARIABLES	
RELAPSE - ADMISSION + CRHTT (%)	
Yes	4997 (33.60)
No	9877 (66.40)
NUMBER OF RELAPSES (whole sample)	
Mean (SD)	1.14 (2.48)
Median (IQR)	0 (0 – 1)
NUMBER OF RELAPSES (those who relapsed)	
Mean (SD)	3.41 (3.24)
Median (IQR)	2 (1 - 4)
DAYS TO RELAPSE (those who relapsed)***	
Mean (SD)	362.6 (284.53)
Median (IQR)	302 (121 – 555)
PSYCHIATRIC ADMISSION	
Yes	3349 (22.52)
No	11525 (77.48)
NUMBER OF ADMISSIONS (whole sample)	
Mean (SD)	0.41 (0.99)
Median (IQR)	0 (0 – 0)
NUMBER OF ADMISSIONS (those admitted)	
Mean (SD)	1.84 (1.33)
Median (IQR)	1 (1 - 2)
DAYS TO ADMISSION (those admitted)***	
Mean (SD)	374.0 (282.40)
Median (IQR)	316(140 - 561)
CRHTT REFERRAL	
Yes	4204 (28.26)
No	10670 (71.74)
NUMBER OF CRHTT REFERRALS (whole sample)	
Mean (SD)	0.73 (1.79)
Median (IQR)	0 (0 – 1)
NUMBER OF CRHTT REFERRALS (those referred)	
Mean (SD)	2.59 (2.55)
Median (IQR)	2 (1 - 3)
DAYS TO CRHTT REFERRAL (those referred)***	
Mean (SD)	398.60 (295.15)
Median (IQR)	358(148 – 602)
DETENTION UNDER MHA	
Yes	2858 (19.21)
No	12016 (80.79)
NUMBER OF DETENTIONS (whole sample)	
Mean (SD)	0.41 (1.06)
Median (IQR)	0 (0 – 0)
NUMBER OF DETENTIONS (those detained)	
Mean (SD)	2.14 (1.48)
Median (IQR)	2 (1 - 3)
DAYS TO DETENTION (those detained)***	
Mean (SD)	390.9 (288.76)
Median (IQR)	340 (150 - 581)
NUMBER OF ED ATTENDANCES (whole sample)	
Mean (SD)	2.23 (5.92)
Median (IQR)	1 (0 – 3)
GENERAL HOSPITAL ADMISSION	
Yes	4468 (30.04)
No	10406 (69.96)
NUMBER OF GENERAL ADMISSIONS (whole sample)	
Mean (SD)	1.40 (3.70)
Median (IQR)	0 (0 - 2)
DEATH	
Yes	201 (1.35)
No	14677 (98.65)
DAYS TO DEATH (those that died)*	
Mean (SD)	495.21 (293.35)
Median (IQR)	462(272 – 633)

This table presents summarised demographics, exposures and outcomes for the cohort (N=14874). Note that for age, inter-quartile range (IQR) is reported rather than full range reported to avoid potentially identifiable data as per ONS SRS requirements. For gender, the ‘Other’ category (containing a very small number of participants) has been combined with ‘Female’, again to avoid potentially identifiable data.

* For time-to-event outcomes, the time of exposure was taken as date of the relevant NCAP audit period for each individual (1st April 2019 or 1st April 2020).

**Table 2:** Associations between exposures and relapse (primary outcome) This table presents the unadjusted and adjusted hazard ratios (HRs) with 95% confidence intervals (CIs) for the primary outcome (relapse, defined as inpatient admission or CRHTT referral). The ‘Full Model’ includes all exposure variables and covariates, while the ‘Final Model’ is based on a refined selection of variables informed by statistical and theoretical considerations. Hazard ratios represent the relative likelihood of relapse occurring at any given time for individuals in one category of a variable compared with the reference category, holding all other variables constant. HR > 1 indicates an increased likelihood of relapse, while HR < 1 indicates a decreased likelihood. Results are adjusted for clustering within services. Results in **bold** indicate p-values ≤0.001 which were considered strong evidence. Results in *italics* indicate p-values between 0.001 and 0.05 which were considered suggestive but not definitive evidence.

Variables	Unadjusted HR (95% CI)	Adjusted HR - Full Model (95% CI)	Adjusted HR - Final Model (95% CI)
Age*			
	**0.98 (0.98–0.98), p<0.001**	0.98 (0.96–1.00), p=0.055	**0.98 (0.98–0.98), p<0.001**
Sex			
Female	Ref	Ref	-
Male	**1.15 (1.08–1.21), p<0.001**	0.94 (0.89–1.00), p=0.054	-
Ethnicity			
White	Ref	Ref	Ref
BAME	**1.25 (1.18–1.33), p<0.001**	**1.11 (1.08–1.15), p<0.001**	**1.18 (1.11–1.26), p<0.001**
Other	*1.13 (1.02–1.24), p=0.020*	1.08 (0.98–1.20), p=0.129	1.09 (0.98–1.20), p=0.108
Patient in employment or education			
No	Ref	Ref	Ref
Yes	**0.90 (0.85–0.96), p=0.001**	**0.73 (0.61–0.88), p=0.001**	*0.92 (0.86-0.97), p =0.003*
Psychiatric admission prior to EIP involvement			
No	Ref	Ref	Ref
Yes	**2.43 (2.29–2.58), p<0.001**	**2.21 (2.06–2.38), p<0.001**	**2.25 (2.11–2.40), p<0.001**
Average care coordinator caseload at treating EIP service*			
	**1.02 (1.01–1.02), p<0.001**	**1.02 (1.01–1.02), p<0.001**	**1.02 (1.01–1.02), p<0.001**
Likelihood that treatment began in <2 weeks (based on proportion meeting waiting time standard at treating service)*			
	1.00 (1.00–1.00), p=0.05	1.00 (0.99–1.02), p=0.751	-
Received Cognitive Behavioural Therapy for Psychosis			
No	Ref	Ref	-
Refused	1.05 (0.97–1.14), p=0.202	1.00 (1.00–1.00), p=0.999	-
Yes	*0.92 (0.85–0.99), p=0.021*	0.95 (0.88–1.03), p=0.199	-
Received Family Intervention			
No	Ref	Ref	-
Refused	*1.07 (1.01–1.15), p=0.031*	0.98 (0.91–1.05), p=0.485	-
Yes	**1.24 (1.15–1.33), p<0.001**	1.05 (0.97–1.14), p=0.191	-
Received carer-focussed intervention			
Not eligible	Ref	Ref	-
No	*1.11 (1.03–1.20), p=0.006*	1.04 (0.96–1.12), p=0.354	-
Yes	**1.28 (1.19–1.37), p<0.001**	*1.09 (1.01–1.18), p=0.021*	-
Received vocational support			
No	Ref	Ref	-
Refused	**1.12 (1.07–1.18), p<0.001**	1.07 (0.99–1.15), p=0.091	-
Yes	**1.23 (1.18–1.29), p<0.001**	*1.09 (1.02–1.17), p=0.013*	-
Received antipsychotic			
No	Ref	Ref	Ref
Yes	**2.20 (2.00–2.41), p<0.001**	**2.30 (1.54–3.43), p<0.001**	**1.32 (1.15–1.52), p<0.001**
Received clozapine			
Not eligible	Ref	Ref	Ref
Not offered	**1.36 (1.25–1.47), p<0.001**	**1.51 (1.36–1.67), p<0.001**	**1.50 (1.36–1.67), p<0.001**
Refused	**1.63 (1.43–1.85), p<0.001**	**1.51 (1.36–1.66), p<0.001**	**1.38 (1.17–1.63), p<0.001**
Yes	**1.74 (1.60–1.90), p<0.001**	0.96 (0.84–1.10), p=0.588	0.97 (0.85–1.10), p=0.614
Received intervention for alcohol cessation			
Not required	Ref	Ref	-
No	**0.85 (0.79–0.91), p<0.001**	1.01 (0.85–1.19), p=0.942	-
Refused	**0.82 (0.75–0.89), p<0.001**	0.91 (0.79–1.05), p=0.211	-
Yes	1.06 (0.98–1.15), p=0.142	*1.19 (1.06–1.33), p=0.002*	-
Received intervention for smoking cessation			
Not required	Ref	Ref	-
No	**0.89 (0.83–0.96), p<0.001**	0.94 (0.81–1.09), p=0.388	-
Refused	1.00 (0.95–1.06), p=0.996	1.00 (0.90–1.11), p=0.959	-
Yes	**1.09 (1.04–1.14), p<0.001**	1.05 (0.97–1.13), p=0.244	-
Received intervention for substance use			
Not required	Ref	Ref	Ref
No	**0.85 (0.79–0.92), p<0.001**	1.04 (0.86–1.25), p=0.703	1.02 (0.91–1.13), p=0.783
Refused	1.01 (0.95–1.09), p=0.697	**1.50 (1.33–1.70), p<0.001**	**1.45 (1.32–1.59), p<0.001**
Yes	**1.17 (1.11–1.23), p<0.001**	**1.47 (1.35–1.59), p<0.001**	**1.52 (1.42–1.63), p<0.001**
Received intervention for weight loss			
Not required	Ref	Ref	-
No	1.02 (0.92–1.14), p=0.695	1.07 (0.91–1.25), p=0.408	-
Refused	1.01 (0.91–1.13), p=0.875	0.91 (0.77–1.08), p=0.277	-
Yes	**1.15 (1.10–1.19), p<0.001**	**0.89 (0.84–0.95), p=0.001**	-
Interaction effect: age x employment**			
AGE : EMPLOYMENT No	NA	Ref	-
AGE : EMPLOYMENT Yes	NA	*1.01 (1.00–1.01), p=0.011*	-
Interaction effect: age x antipsychotic**			
AGE : ANTIPSYCHOTIC No	NA	Ref	-
AGE : ANTIPSYCHOTIC Yes	NA	*0.98 (0.97–0.99), p=0.002*	-

* For the continuous variables ‘age’, ‘care coordinator caseload’ and ‘proportion meeting waiting time standard’, stated hazard ratios indicate the change in hazard with a one-unit increase in the exposure. For example, each additional person on a care coordinator’s caseload increased the hazard of relapse by 2% (HR 1.02, 95% CI 1.01–1.02, p<0.001).

** Interaction effects indicate the change in the hazard ratio for the second variable for each unit of change in the first. For example, for individuals taking antipsychotic medication, each additional year of age decreases the hazard rate by 2% compared with those not taking it. This suggests that the increased hazard rate of relapse associated with antipsychotic medication reduces with age.

## Data Availability

RW, EP, AP and AB had access to the full study dataset. The dataset and code script for analysis is held in the Office for National Statistics’ Secure Research Service and as such it is unfortunately not possible to share on request. Statistical data from the ONS is subject to Crown Copyright. The use of the ONS statistical data in this work does not imply the endorsement of the ONS in relation to the interpretation or analysis of the statistical data. This work uses research datasets which may not exactly reproduce National Statistics aggregates.
