# Supplementary material for: Critical components of ‘Early Intervention in Psychosis’: a national retrospective cohort study
Source: Br J Psychiatry. Author manuscript; Available in PMC 2025 Nov 27. (PMC7617821; doi:10.1192/bjp.2025.126)
Supplement: Supplementary Material [file EMS204643-supplement-Supplementary_Material.pdf]

CRITICAL COMPONENTS OF ‘EARLY INTERVENTION IN PSYCHOSIS: A NATIONAL RETROSPECTIVE COHORT STUDY

Ryan Williams, Ed Penington, Veenu Gupta, Alan Quirk, Apostolos Tsiachristas, Michelle Rickett, Caroline Chew-Graham, David Shiers, Paul French, Belinda Lennox, Alex Bottle, Mike J Crawford

Supplementary Appendix

INDEX

1. CHANGES FROM THE PUBLISHED PROTOCOL
2. STROBE CHECKLIST
3. DETAILED STATISTICAL METHODS
4. COHORT DEMOGRAPHICS
5. EXPOSURE VARIABLES
6. OUTCOME VARIABLES
7. RESULTS – SECONDARY OUTCOMES
8. MISSING DATA
9. SENSITIVITY ANALYSES

CHANGES FROM THE PUBLISHED PROTOCOL

1. In the published protocol[1] we indicated we would only have access to data from the 2019-20 round of the NCAP (10,560 individuals) – however, we were fortunately able to also access data from the 2020-2021 round of the NCAP, increasing the total sample size to 14,874 once duplicates were removed (individuals who had been sampled in both rounds of the NCAP).

2. In the published protocol we indicated we would also include an additional exposure variable - whether patients were monitoring with validated outcome measures (such as HONOS). However, on accessing the data there were large amounts of missing data for these outcome measures which appeared to be missing Not At Random, so they were dropped from the analysis.

STROBE CHECKLIST

STROBE Statement[2] —Checklist of items that should be included in reports of ***cohort studies***

	Item No	Recommendation	Reported on page
**Title and abstract**	1	(*a*) Indicate the study's design with a commonly used term in the title or the abstract	p.1
		(*b*) Provide in the abstract an informative and balanced summary of what was done and what was found	p.2
**Introduction**			
Background/rationale	2	Explain the scientific background and rationale for the investigation being reported	p.3
Objectives	3	State specific objectives, including any prespecified hypotheses	p.3
**Methods**			
Study design	4	Present key elements of study design early in the paper	p.3-4
Setting	5	Describe the setting, locations, and relevant dates, including periods of recruitment, exposure, follow-up, and data collection	p.3-4
Participants	6	(*a*) Give the eligibility criteria, and the sources and methods of selection of participants. Describe methods of follow-up	p.3-4
		(*b*) For matched studies, give matching criteria and number of exposed and unexposed	N/A
Variables	7	Clearly define all outcomes, exposures, predictors, potential confounders, and effect modifiers. Give diagnostic criteria, if applicable	p.4
Data sources/ measurement	8*	For each variable of interest, give sources of data and details of methods of assessment (measurement). Describe comparability of assessment methods if there is more than one group	p.4
Bias	9	Describe any efforts to address potential sources of bias	p.4

Study size	10	Explain how the study size was arrived at	Appendix p.7
Quantitative variables	11	Explain how quantitative variables were handled in the analyses. If applicable, describe which groupings were chosen and why	p.4, Appendix p.6-7
Statistical methods	12	(*a*) Describe all statistical methods, including those used to control for confounding	p.4, Appendix p.6-7
		(*b*) Describe any methods used to examine subgroups and interactions	p.4, Appendix p.6-7
		(*c*) Explain how missing data were addressed	p.6, Appendix p.29-32
		(*d*) If applicable, explain how loss to follow-up was addressed	N/A
		(*e*) Describe any sensitivity analyses	Appendix p.33-36
**Results**			
Participants	13*	(a) Report numbers of individuals at each stage of study—e.g. numbers potentially eligible, examined for eligibility, confirmed eligible, included in the study, completing follow-up, and analysed	p.4
		(b) Give reasons for non-participation at each stage	N/A
		(c) Consider use of a flow diagram	N/A
Descriptive data	14*	(a) Give characteristics of study participants (e.g. demographic, clinical, social) and information on exposures and potential confounders	p.12-12
		(b) Indicate number of participants with missing data for each variable of interest	Appendix p.30-31
		(c) Summarise follow-up time (e.g. average and total amount)	p.4
Outcome data	15*	Report numbers of outcome events or summary measures over time	p.13-14
Main results	16	(*a*) Give unadjusted estimates and, if applicable, confounder-adjusted estimates and their precision (e.g. 95% confidence interval). Make clear which confounders were adjusted for and why they were included	p.15-16, Appendix p.13-28

		(*b*) Report category boundaries when continuous variables were categorized	N/A
		(*c*) If relevant, consider translating estimates of relative risk into absolute risk for a meaningful time period	p.17-18
Other analyses	17	Report other analyses done—e.g. analyses of subgroups and interactions, and sensitivity analyses	Appendix p.33-36
**Discussion**			
Key results	18	Summarise key results with reference to study objectives	p.4-6
Limitations	19	Discuss limitations of the study, taking into account sources of potential bias or imprecision. Discuss both direction and magnitude of any potential bias	p.8
Interpretation	20	Give a cautious overall interpretation of results considering objectives, limitations, multiplicity of analyses, results from similar studies, and other relevant evidence	p.6-8
Generalisability	21	Discuss the generalisability (external validity) of the study results	p.8
**Other information**			
Funding	22	Give the source of funding and the role of the funders for the present study and, if applicable, for the original study on which the present article is based	p.9

*Give information separately for exposed and unexposed groups.

Note: An Explanation and Elaboration article discusses each checklist item and gives methodological background and published examples of transparent reporting. The STROBE checklist is best used in conjunction with this article (freely available on the Web sites of PLoS Medicine at http://www.plosmedicine.org/, Annals of Internal Medicine at http://www.annals.org/, and Epidemiology at http://www.epidem.com/). Information on the STROBE Initiative is available at http://www.strobe-statement.org.

DETAILED STATISTICAL METHODS

All analyses were performed using 'R'.[3] We conducted a comprehensive analysis of the associations between exposure variables and primary and secondary outcomes while accounting for potential confounding factors.

Initially, we generated descriptive statistics for all exposure variables, outcome measures and covariates. We used unadjusted tests to explore relationships between the exposure variables, covariates and primary and secondary outcomes.

Next, for each outcome we fit an adjusted (multivariable) regression model including all exposures and covariates identified as potentially significant from our a priori planning (see DAG below) – to consider the simultaneous (rather than isolated) effect of all variables, taking potential confounding effects into account. We used multilevel regression models to account for clustering effects (as patients in the cohort are grouped within EIP services). We included a random intercept for EIP team to account for potential team-level variations, and random slopes between EIP team and NCAP audit round to account for yearly differences e.g. due to COVID-19. This approach acknowledges the potential correlation between individuals within the same service, ensuring appropriate adjustments were made to obtain unbiased estimates. It also allows service-level effects to be handled properly.[4]

Finally, we built refined statistical models for each analysis on the basis of the following pragmatic principles:

- Explanatory variables hypothesised to be associated with outcomes from a-priori discussions and previous studies were accounted for in model exploration;
- Demographic variables were included in model exploration;
- Interactions were checked and included in model exploration if influential;
- Final model selection was performed using a 'criterion-based' approach, aiming to minimise the Akaike Information Criterion (AIC) to establish models that captured as much information in the data with as few parameters as possible.[5]

We examined associations between exposures and 'time to event' outcomes (relapse as indicated by admission or referral to CRHTT; detention under mental health act; mortality) using Cox regression. Cox regression allows for the analysis of time-to-event data while accommodating censoring effects, which occurred if participants had not experienced the event of interest during the study period. The time of exposure was taken as the end of the relevant NCAP audit period for each individual (October 2019 or 2020).

We anticipated a strong association between some of our time-to-event outcomes (specifically time to referral to CRHTT, psychiatric admission, and detention under the Mental Health Act). Our use of referral to CRHTT and psychiatric admission specifically as proxy measures of 'relapse' to assess the time from exposures to this event is an approach that has been widely used elsewhere.[6-8] To evaluate the association between these outcomes, we examined the concordance between survival probabilities. We also examined psychiatric admission and CRHTT referral separately as secondary outcomes to identify any deviance in patterns between these outcomes.

We examined associations between exposures and 'count' outcomes (e.g. number of general hospital admissions and emergency department attendances) using zero-inflated negative binomial regression, in order to account for the overdispersion observed in the data and the fact that a large proportion of individuals did not experience each outcome. Offset terms were added to the models to account for the fact that individuals had different 'observation' durations due to participating in different rounds of the NCAP (those from 2020 NCAP only effectively had 1-year shorter follow-up period).

Given the large number of comparisons performed, we adopted a more stringent significance threshold ($p \leq 0.001$) to reduce the likelihood of spurious findings while maintaining a balance between Type I and Type II error rates. This threshold was chosen as a heuristic to focus on robust associations rather than applying formal corrections, which may be overly conservative in observational analyses. Results with p-values between 0.001 and 0.05 were considered suggestive but not definitive. This threshold of $p \leq 0.001$ is broadly consistent with the significance levels used when controlling the false discovery rate at 5% in large datasets.[9]

We assessed model assumptions for Cox regression models using Schoenfeld residuals, both statistically via the *cox.zph* function in R and visually through plots of residuals over time. For negative binomial models, we evaluated overdispersion using the dispersion parameter (theta) and inspected deviance residuals to assess goodness-of-fit. Across

all models, diagnostic plots (residuals versus fitted values) were used to identify potential model misspecifications, and influential points were assessed using Cook's distance and leverage plots. Multicollinearity was checked using variance inflation factors (VIF) for all covariates. Unfortunately, plots of residuals were considered potentially identifiable data and were not cleared for extraction from the ONS secure research service environment.

Prior to the study initiation we estimated that our regression models would be adequately powered to detect small differences in the likelihood of our primary outcome for all exposures and eliminate bias in the regression coefficients, based on the widely-used standard of 20 outcomes per exposure variable, derived from previous studies.[10]

Figure A1 – Directed Acyclic Graph

In preparation for this analysis, we constructed a Directed Acyclic Graph (DAG)[4] to visually represent hypothesised causal relationships among the variables and covariates in our data (as well as potential unobserved mediators/ confounders), in order to guide inclusion in regression models. This process was informed based on the theoretical expertise of co-authors (including experts in this field and experts by experience) and previous research evidence. We included participant age, sex, ethnicity, employment status, and whether their referral to an EIP service had been preceded by an inpatient admission (a proxy marker of overall severity of initial presentation).

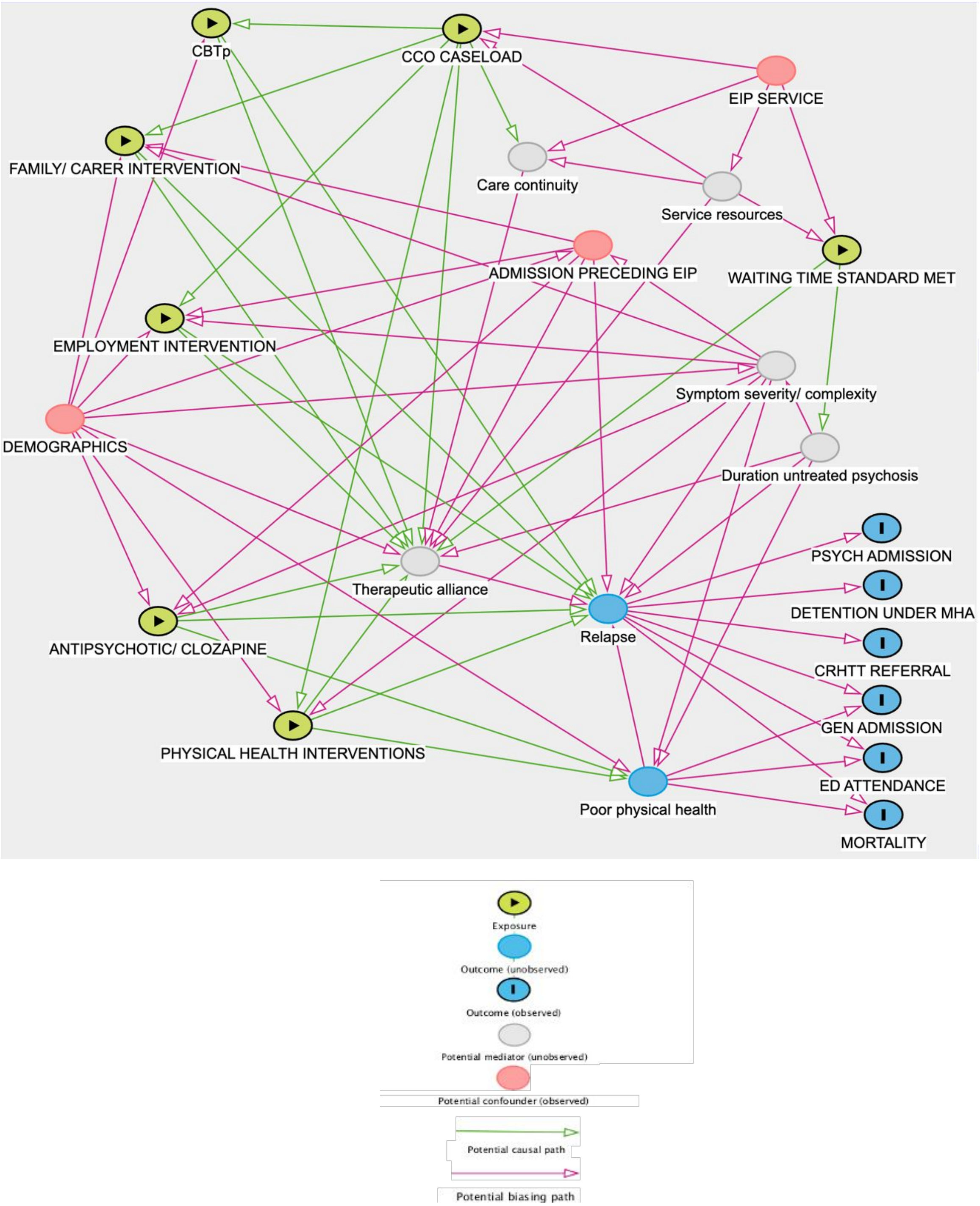

COHORT DEMOGRAPHICS

Figure A2 – Ethnicity breakdown

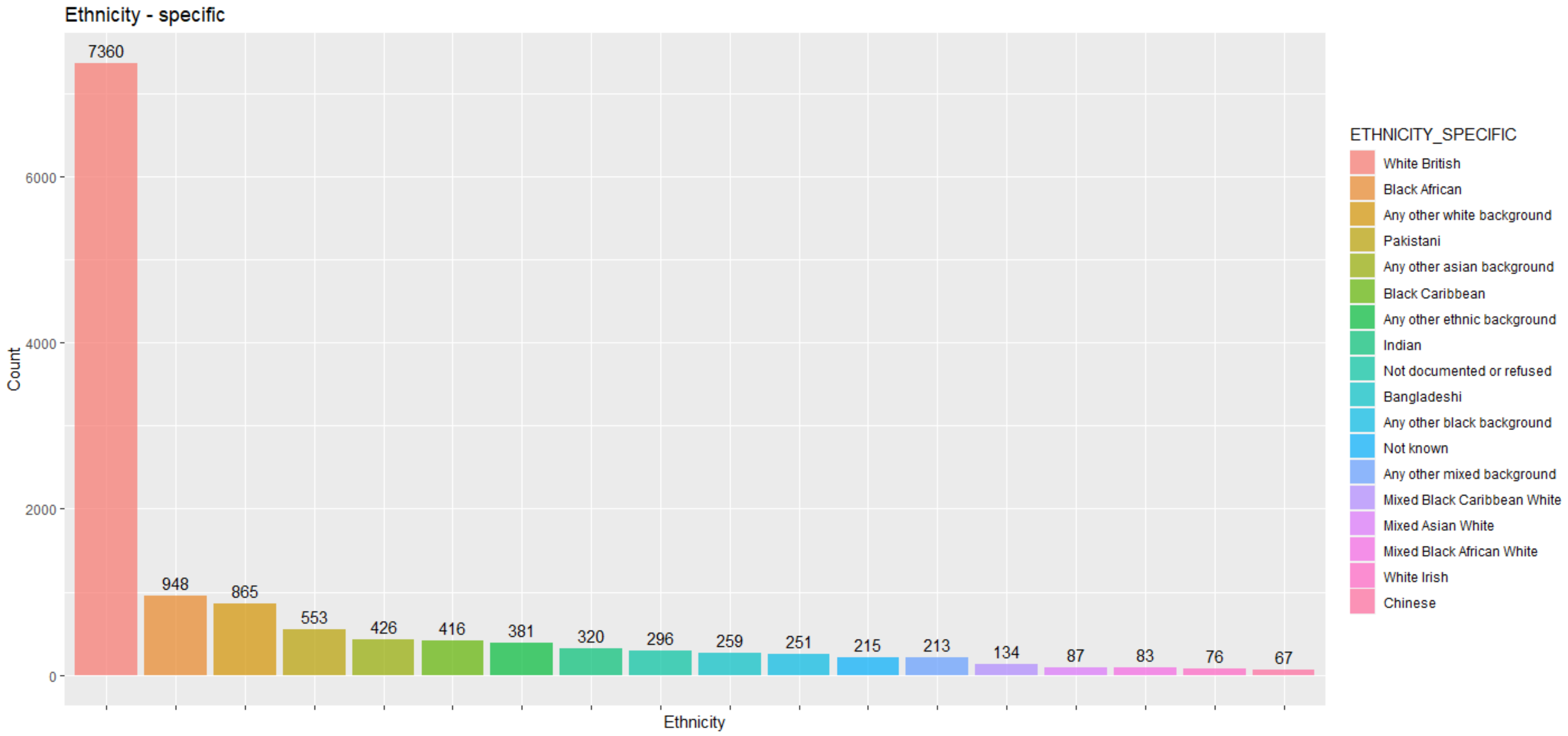

Figure A3 – Comorbidity distribution by demographics

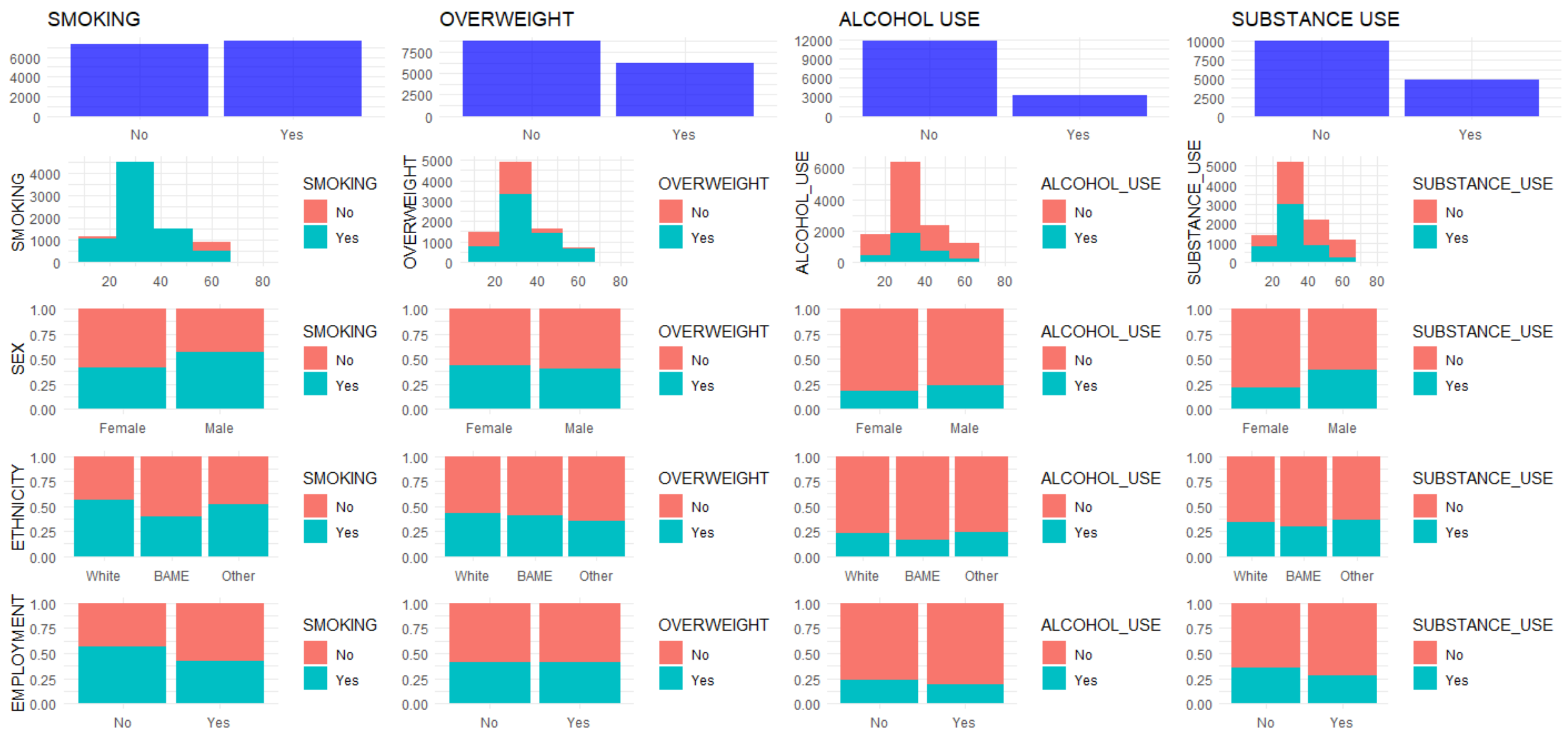

Table A1 – Comorbidity breakdown by demographics

N = 14874	SMOKING (%)		OVERWEIGHT (%)		ALCOHOL USE (%)		SUBSTANCE USE (%)		PREVIOUS ADMISSION BEFORE EI (%)	
	Yes	No	Yes	No	Yes	No	Yes	No	Yes	No
TOTAL	7596 (51.1)	7278 (48.9)	6144 (41.3)	8730 (58.7)	3192 (21.5)	11682 (78.5)	4869 (32.7)	10005 (67.3)	8228 (55.3)	6646 (44.7)
AGE										
Mean (SD)	32.7 (10.4)	34.4 (12.4)	34.7 (11.7)	32.7 (11.2)	33.0 (10.7)	33.7 (11.6)	31.2 (9.6)	34.6 (12.1)	32.9 (11.0)	34.3 (12.0)
Median (IQR)	30 (25-38)	31 (24-42)	32 (25-42)	30 (24-38)	31 (25-39)	30 (25-40)	29 (24-36)	32 (25-42)	30 (24-38)	31 (25-41)
GENDER (%)										
Male	5284 (57.3)	3941 (42.7)	3680 (39.9)	5545 (60.1)	2202 (23.9)	7023 (76.1)	3647 (39.5)	5578 (60.5)	5176 (56.1)	4049 (43.9)
Female (or other)*	2312 (40.9)	3337 (59.1)	2464 (43.6)	3185 (56.4)	990 (17.5)	4659 (82.5)	1222 (21.6)	4427 (78.4)	3052 (54.0)	2597 (46.0)
ETHNICITY (%)										
White	5374 (55.8)	4253 (44.2)	4090 (42.5)	5537 (57.5)	2230 (23.2)	7397 (76.8)	3244 (33.7)	6383 (66.3)	5176 (53.8)	4451 (46.2)
BAME	1557 (39.2)	2416 (60.8)	1613 (40.6)	2360 (59.4)	657 (16.5)	3316 (83.5)	1161 (29.2)	2812 (70.8)	2363 (59.5)	1611 (40.5)
Other	665 (52.2)	609 (47.8)	441 (34.6)	833 (65.4)	305 (23.9)	969 (76.1)	464 (36.4)	810 (63.6)	690 (54.2)	584 (45.8)
EMPLOYMENT(%)										
No	5068 (56.6)	3885 (43.4)	4917 (56.3)	3813 (43.7)	2068 (23.1)	6885 (76.9)	3226 (36.0)	5727 (64.0)	5085 (56.8)	3868 (43.2)
Yes	2528 (42.7)	3393 (57.2)	2679 (43.6)	3465 (56.4)	1124 (19.0)	4797 (81.0)	1643 (27.7)	4278 (72.3)	3143 (53.1)	2778 (46.9)

* ‘Other’ category <10, categories combined to avoid potentially identifiable data as per ONS SRS requirements

EXPOSURE VARIABLES

Figure A4 – Exposure variable distribution

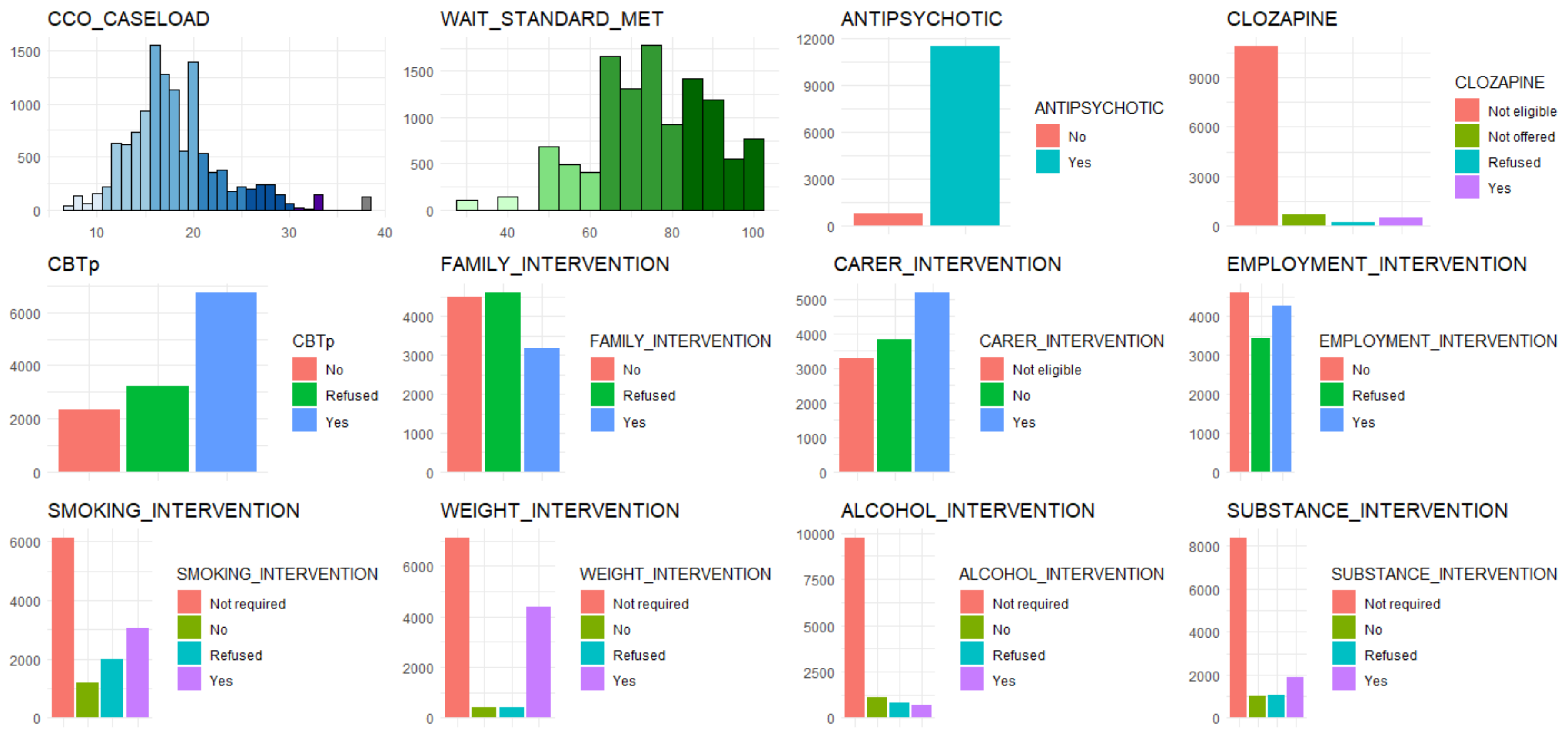

Table A2 – Exposure variable distribution

	N = 14874
SERVICE LEVEL VARIABLES	
CARE COORDINATOR CASELOAD	
Mean (SD)	18.5 (5.7)
Min-Max	7.0 – 54.5
Median (IQR)	17.4 (15.1 – 20.4)
PROPORTION WAITING TIME STANDARD MET	
Mean (SD)	74.7 (14.4)
Min-Max	30 - 100
Median (IQR)	75.0 (64.0 – 86.0)
PATIENT LEVEL VARIABLES	
RECEIVED ANTIPSYCHOTIC (%)	
No	924 (6.2)
Yes	13950 (93.8)
RECEIVED CLOZAPINE (%)	
Not eligible	12964 (87.2)
Not offered	987 (6.7)
Refused	291 (1.9)
Yes	630 (4.2)
RECEIVED CBTp (%)	
No	3646 (24.5)
Refused	3975 (26.7)
Yes	7253 (48.8)
RECEIVED FAMILY INTERVENTION (%)	
No	6033 (40.6)
Refused	5508 (37.0)
Yes	3333 (22.4)
RECEIVED CARER INTERVENTION (%)	
Not eligible	4021 (27.0)
No	4724 (31.8)
Yes	6129 (41.2)
RECEIVED EMPLOYMENT INTERVENTION (%)	
No	5838 (39.2)
Refused	4160 (28.0)
Yes	4876 (32.8)
RECEIVED SMOKING INTERVENTION (%)	
Not required	7278 (48.9)
No	1512 (10.2)
Refused	2386 (16.0)
Yes	3698 (24.9)
RECEIVED WEIGHT INTERVENTION (%)	
Not required	8730 (58.7)
No	487 (3.3)
Refused	465 (3.1)
Yes	5192 (34.9)
RECEIVED ALCOHOL INTERVENTION (%)	
Not required	11682 (78.5)
No	1377 (9.3)
Refused	975 (6.6)
Yes	840 (5.6)
RECEIVED SUBSTANCE INTERVENTION (%)	
Not required	10005 (67.3)
No	1243 (8.4)
Refused	1327 (8.9)
Yes	2299 (15.4)

OUTCOME VARIABLES

Table A3 – Outcome variable distribution

	N = 14874
RELAPSE - ADMISSION + CRHTT (%)	
Yes	4997 (33.60)
No	9877 (66.40)
NUMBER OF RELAPSES (whole sample)	
Mean (SD)	1.14 (2.48)
Median (IQR)	0 (0 – 1)
NUMBER OF RELAPSES (those who relapsed)	
Mean (SD)	3.41 (3.24)
Median (IQR)	2 (1 - 4)
TIME TO RELAPSE (those who relapsed)	
Mean (SD)	362.6 (284.53)
Median (IQR)	302 (121 – 555)
ADMISSION	
Yes	3349 (22.52)
No	11525 (77.48)
NUMBER OF ADMISSIONS (whole sample)	
Mean (SD)	0.41 (0.99)
Median (IQR)	0 (0 – 0)
NUMBER OF ADMISSIONS (those admitted)	
Mean (SD)	1.84 (1.33)
Median (IQR)	1 (1 - 2)
TIME TO ADMISSION (those admitted)	
Mean (SD)	374.0 (282.40)
Median (IQR)	316 (140 - 561)
CRHTT REFERRAL	
Yes	4204 (28.26)
No	10670 (71.74)
NUMBER OF CRHTT REFERRALS (whole sample)	
Mean (SD)	0.73 (1.79)
Median (IQR)	0 (0 – 1)
NUMBER OF CRHTT REFERRALS (those referred)	
Mean (SD)	2.59 (2.55)
Median (IQR)	2 (1 - 3)
TIME TO CRHTT REFERRAL (those referred)	
Mean (SD)	398.60 (295.15)
Median (IQR)	358 (148 - 602)
DETENTION	
Yes	2858 (19.21)
No	12016 (80.79)
NUMBER OF DETENTIONS (whole sample)	
Mean (SD)	0.41 (1.06)
Median (IQR)	0 (0 – 0)
NUMBER OF DETENTIONS (those detained)	
Mean (SD)	2.14 (1.48)
Median (IQR)	2 (1 - 3)
TIME TO DETENTION (those detained)	
Mean (SD)	390.9 (288.76)
Median (IQR)	340 (150 - 581)
NUMBER OF ED ATTENDANCES (whole sample)	
Mean (SD)	2.23 (5.92)
Median (IQR)	1 (0 – 3)
GENERAL ADMISSION	
Yes	4468 (30.04)
No	10406 (69.96)
NUMBER OF GENERAL ADMISSIONS (whole sample)	
Mean (SD)	1.40 (3.70)
Median (IQR)	0 (0 - 2)
DEATH	
Yes	201 (1.35)
No	14677 (98.65)
TIME TO DEATH (those that died)	
Mean (SD)	495.21 (293.35)
Median (IQR)	462 (272 – 633)

ANALYSIS – SECONDARY OUTCOMES

Table A4 – Psychiatric admission

This table presents the unadjusted and adjusted hazard ratios (HRs) with 95% confidence intervals (CIs) for the secondary outcome ‘psychiatric inpatient admission’. The ‘Full Model’ includes all exposure variables and covariates, while the ‘Final Model’ is based on a refined selection of variables informed by statistical and theoretical considerations. Hazard ratios represent the relative likelihood of relapse occurring at any given time for individuals in one category of a variable compared with the reference category, holding all other variables constant. HR > 1 indicates an increased likelihood of relapse, while HR < 1 indicates a decreased likelihood. Results are adjusted for clustering within services. Results in **bold** indicate p-values ≤0.001 which were considered strong evidence. Results in *italics* indicate p-values between 0.001 and 0.05 which were considered suggestive but not definitive evidence.

Variables	Unadjusted HR (95% CI)	Adjusted HR - Full Model (95% CI)	Adjusted HR - Final Model (95% CI)
Age*			
	**0.97 (0.97–0.98), p<0.001**	1.00 (0.99–1.02), p=0.740	**0.98 (0.97–0.98), p<0.001**
Sex			
Female	Ref	Ref	-
Male	**1.22 (1.14–1.31), p<0.001**	0.96 (0.89–1.03), p=0.261	-
Ethnicity			
White	Ref	Ref	Ref
BAME	**1.45 (1.34–1.56), p<0.001**	**1.71 (1.33–2.21), p<0.001**	**1.32 (1.22–1.42), p<0.001**
Other	**1.23 (1.09–1.39), p=0.001**	*1.54 (1.01–2.36), p=0.047*	*1.16 (1.03–1.30), p=0.017*
Patient in employment or education			
No	Ref	Ref	Ref
Yes	**0.85 (0.79–0.91), p<0.001**	**0.68 (0.54–0.87), p<0.001**	**0.87 (0.81–0.94), p<0.001**
Psychiatric admission prior to EIP involvement			
No	Ref	Ref	Ref
Yes	**3.65 (3.36–3.96), p<0.001**	**3.22 (2.96–3.51), p<0.001**	**3.29 (3.03-3.58), p<0.001**
Average care coordinator caseload at treating EIP service*			
	**1.02 (1.01–1.02), p<0.001**	**1.02 (1.01–1.02), p<0.001**	**1.02 (1.01–1.02), p<0.001**
Likelihood that treatment began in <2 weeks (based on proportion meeting waiting time standard at treating service)*			
	1.00 (0.99–1.00), p=0.080	1.00 (1.00–1.00), p=0.213	-
Received Cognitive Behavioural Therapy for Psychosis			
No	Ref	Ref	Ref
Refused	*1.11 (1.01–1.22), p=0.030*	1.04 (0.94–1.15), p=0.495	1.05 (0.95-1.16), p =0.324
Yes	**0.86 (0.78–0.94), p=0.001**	0.94 (0.86–1.03), p=0.160	*0.91 (0.83-1.00), p =0.057*
Received Family Intervention			
No	Ref	Ref	-
Refused	*1.08 (1.00–1.17), p=0.047*	0.97 (0.89–1.06), p=0.505	-
Yes	**1.22 (1.12–1.33), p<0.001**	1.05 (0.97–1.15), p=0.355	-
Received carer-focussed intervention			
Not eligible	Ref	Ref	-
No	1.09 (0.99–1.19), p=0.076	0.98 (0.90–1.08), p=0.725	-
Yes	**1.25 (1.14–1.35), p<0.001**	1.02 (0.94–1.12), p=0.613	-
Received vocational support			
No	Ref	Ref	-
Refused	**1.22 (1.12–1.33), p<0.001**	1.07 (0.97–1.17), p=0.162	-
Yes	**1.33 (1.23–1.44), p<0.001**	*1.09 (1.01–1.19), p=0.034*	-
Received antipsychotic			
No	Ref	Ref	Ref
Yes	**2.08 (1.74–2.49), p<0.001**	**3.09 (1.81–5.27), p<0.001**	**1.41 (1.17–1.68), p<0.001**
Received clozapine			
Not eligible	Ref	Ref	Ref
Not offered	**1.81 (1.61–2.04), p<0.001**	**1.56 (1.38–1.75), p<0.001**	**1.55 (1.37–1.74), p<0.001**
Refused	**2.01 (1.66–2.42), p<0.001**	**1.44 (1.19–1.75), p<0.001**	**1.46 (1.21–1.76), p<0.001**
Yes	**1.49 (1.28–1.73), p<0.001**	1.01 (0.86–1.17), p=0.919	1.01 (0.86–1.17), p=0.932
Received intervention for alcohol cessation			
Not required	Ref	Ref	-
No	0.93 (0.82–1.04), p=0.213	0.96 (0.79–1.17), p=0.713	-
Refused	1.04 (0.91–1.19), p=0.528	0.86 (0.72–1.02), p=0.079	-

Yes	**1.27 (1.11–1.45), p<0.001**	1.11 (0.97–1.27), p=0.138	-
Received intervention for smoking cessation			
Not required	Ref	Ref	-
No	0.98 (0.87–1.11), p=0.723	*0.78 (0.65–0.93), p=0.007*	-
Refused	**1.29 (1.17–1.42), p<0.001**	1.07 (0.95–1.19), p=0.268	-
Yes	**1.37 (1.26–1.48), p<0.001**	*1.10 (1.00–1.20), p=0.045*	-
Received intervention for substance use			
Not required	Ref	Ref	Ref
No	1.06 (0.93–1.21), p=0.370	1.34 (0.85–2.12), p=0.210	1.12 (0.99–1.28), p=0.077
Refused	**1.56 (1.40–1.74), p<0.001**	**2.02 (1.35–3.01), p=0.001**	**1.53 (1.37–1.71), p<0.001**
Yes	**1.82 (1.68–1.98), p<0.001**	*1.67 (1.21–2.30), p=0.002*	**1.56 (1.43–1.70), p<0.001**
Received intervention for weight loss			
Not required	Ref	Ref	-
No	0.99 (0.82–1.19), p=0.902	1.03 (0.86–1.24), p=0.743	-
Refused	0.88 (0.72–1.08), p=0.218	0.94 (0.76–1.15), p=0.519	-
Yes	**0.85 (0.79–0.92), p<0.001**	**0.85 (0.79–0.92), p<0.001**	-
AGE x ETHNICITY			
AGE : ETHNICITY White	NA	Ref	-
AGE : ETHNICITY BAME	NA	*0.99 (0.98-0.99), p=0.037*	-
AGE : ETHNICITY Other	NA	0.99 (0.98-1.00), p=0.177	-
AGE x EMPLOYMENT			
AGE : EMPLOYMENT No	NA	Ref	-
AGE : EMPLOYMENT Yes	NA	*1.01 (1.00-1.02), p=0.017*	-
AGE x ANTIPSYCHOTIC			
AGE : ANTIPSYCHOTIC No	NA	Ref	-
AGE : ANTIPSYCHOTIC Yes	NA	**0.98 (0.96-0.99), p=0.001**	-

* For the continuous variables ‘age’, ‘care coordinator caseload’ and ‘proportion meeting waiting time standard’, stated hazard ratios indicate the change in hazard with a one-unit increase in the exposure. For example, each additional person on a care coordinator's caseload increased the hazard of admission by 2% (HR 1.02, 95% CI 1.01–1.02, p<0.001).

** Interaction effects indicate the change in the hazard ratio for the second variable for each unit of change in the first. For example, for individuals taking antipsychotic medication, each additional year of age decreases the hazard rate by 2% compared with those not taking it. This suggests that the increased hazard rate of admission associated with antipsychotic medication reduces with age.

Table A5 – Duration admission

This table presents the unadjusted and adjusted incidence rate ratios (IRRs) with 95% confidence intervals (CIs) for the count outcome ‘bed days of admission’ (over follow-up period). The ‘Full Model’ includes all exposure variables and covariates, while the ‘Final Model’ is based on a refined selection of variables informed by statistical and theoretical considerations. Incidence rate ratios therefore represent the relative duration of time spent admitted for individuals in one category of a variable compared with the reference category, holding all other variables constant. IRR > 1 indicates an increased duration, while IRR < 1 indicates a decreased rate. Results are adjusted for clustering within services. Results in **bold** indicate p-values ≤0.001, considered strong evidence. Results in *italics* indicate p-values between 0.001 and 0.05, considered suggestive but not definitive evidence.

Variables	Unadjusted Mean Difference (95% CI)	Adjusted Mean Difference - Full Model (95% CI)	Adjusted Mean Difference - Final Model (95% CI)
Age*			
	**0.96 (0.95–0.97), p<0.001**	1.01 (0.98–1.03), p=0.672	**0.97 (0.96–0.98), p<0.001**
Sex			
Female	Ref	Ref	-
Male	**1.42 (1.20–1.68), p<0.001**	0.97 (0.82–1.14), p=0.684	-
Ethnicity			
White	Ref	Ref	Ref
BAME	**1.41 (1.17–1.70), p<0.001**	**2.15 (1.25–3.69), p=0.009**	*1.30 (1.08–1.56), p=0.004*
Other	*1.40 (1.05–1.91), p=0.025*	*2.75 (1.01–7.43), p=0.028*	1.18 (0.90–1.59), p=0.243
Patient in employment or education			
No	Ref	Ref	Ref
Yes	**0.73 (0.62–0.86), p<0.001**	0.74 (0.45–1.22), p=0.233	**0.76 (0.65–0.90), p=0.001**
Psychiatric admission prior to EIP involvement			
No	Ref	Ref	Ref
Yes	**5.32 (4.54–6.23), p<0.001**	**4.66 (3.96–5.48), p<0.001**	**4.54 (3.87–5.33), p<0.001**
Average care coordinator caseload at treating EIP service*			
	**1.02 (1.01–1.04), p=0.001**	*1.01 (1.00–1.02), p=0.015*	*1.01 (1.00–1.02), p=0.022*
Likelihood that treatment began in <2 weeks (based on proportion meeting waiting time standard at treating service)*			
	1.00 (0.99–1.00), p=0.205	1.00 (0.99–1.00), p=0.585	-
Received Cognitive Behavioural Therapy for Psychosis			
No	Ref	Ref	Ref
Refused	0.94 (0.73–1.20), p=0.615	*0.76 (0.59–0.97), p=0.026*	*0.84 (0.66–0.96), p=0.037*
Yes	**0.68 (0.55–0.85), p=0.001**	**0.67 (0.53–0.84), p<0.001**	**0.73 (0.59–0.89), p=0.002**
Received Family Intervention			
No	Ref	Ref	-
Refused	1.15 (0.96–1.39), p=0.138	0.94 (0.77–1.15), p=0.554	-
Yes	1.22 (0.99–1.51), p=0.061	1.00 (0.80–1.26), p=0.966	-
Received carer-focussed intervention			
Not eligible	Ref	Ref	-
No	1.06 (0.85–1.31), p=0.602	1.09 (0.88–1.34), p=0.417	-
Yes	*1.27 (1.03–1.55), p=0.021*	1.09 (0.88–1.35), p=0.399	-
Received vocational support			
No	Ref	Ref	-
Refused	*1.24 (1.01–1.52), p=0.041*	1.18 (0.96–1.46), p=0.112	-
Yes	*1.35 (1.11–1.63), p=0.002*	*1.21 (1.00–1.47), p=0.047*	-
Received antipsychotic			
No	Ref	Ref	Ref
Yes	**2.51 (1.79–3.41), p<0.001**	*4.24 (1.77–10.04), p=0.002*	1.36 (0.98–1.84), p=0.054
Received clozapine			
Not eligible	Ref	Ref	Ref
Not offered	**2.02 (1.45–2.92), p<0.001**	*1.44 (1.05–2.05), p=0.030*	*1.47 (1.07–2.08), p=0.024*
Refused	*2.51 (1.47–4.79), p=0.002*	1.75 (1.04–3.22), p=0.049	*1.67 (1.00–3.08), p=0.053*
Yes	**2.06 (1.53–3.50), p<0.001**	1.14 (0.53–2.15), p=0.100	1.10 (0.58–2.25), p=0.110
Received intervention for alcohol cessation			
Not required	Ref	Ref	-
No	0.86 (0.66–1.16), p=0.304	0.82 (0.53–1.31), p=0.400	-
Refused	1.11 (0.81–1.57), p=0.549	0.89 (0.57–1.40), p=0.583	-
Yes	*1.53 (1.09–2.23), p=0.018*	1.27 (0.91–1.83), p=0.170	-
Received intervention for smoking cessation			
Not required	Ref	Ref	-
No	1.02 (0.78–1.37), p=0.871	*0.53 (0.35–0.81), p=0.002*	-

Refused	**1.48 (1.18–1.88), p=0.001**	1.09 (0.83–1.43), p=0.539	-
Yes	**1.61 (1.32–1.97), p<0.001**	1.16 (0.94–1.43), p=0.162	-
Received intervention for substance use			
Not required	NA	NA	Ref
No	1.14 (0.86–1.55), p=0.385	2.25 (0.81–6.39), p=0.105	1.22 (0.93–1.65), p=0.164
Refused	**1.77 (1.34–2.39), p<0.001**	1.51 (0.62–3.70), p=0.389	**1.44 (1.10–1.93), p=0.010**
Yes	**2.10 (1.68–2.65), p<0.001**	*3.16 (1.48–6.66), p=0.003*	**1.68 (1.35–2.12), p<0.001**
Received intervention for weight loss			
Not required	NA	NA	-
No	0.67 (0.44–1.11), p=0.095	*0.60 (0.40–0.97), p=0.024*	-
Refused	0.86 (0.56–1.44), p=0.545	1.02 (0.66–1.66), p=0.933	-
Yes	0.85 (0.71–1.01), p=0.065	*0.78 (0.65–0.93), p=0.005*	-

* For the continuous variables ‘age’, ‘care coordinator caseload’ and ‘proportion meeting waiting time standard’, stated incidence ratios indicate the change in incidence rate with a one-unit increase in the exposure. For example, each additional year of age reduced the incidence rate of bed days by 3% (IR 0.97, 95% CI 0.96–0.98, p<0.001).

Table A6 – CRHTT referral

This table presents the unadjusted and adjusted hazard ratios (HRs) with 95% confidence intervals (CIs) for the secondary outcome 'CRHTT referral'. The 'Full Model' includes all exposure variables and covariates, while the 'Final Model' is based on a refined selection of variables informed by statistical and theoretical considerations. Hazard ratios represent the relative likelihood of relapse occurring at any given time for individuals in one category of a variable compared with the reference category, holding all other variables constant. HR > 1 indicates an increased likelihood of relapse, while HR < 1 indicates a decreased likelihood. Results are adjusted for clustering within services. Results in **bold** indicate p-values ≤0.001 which were considered strong evidence. Results in *italics* indicate p-values between 0.001 and 0.05 which were considered suggestive but not definitive evidence.

Variables	Unadjusted HR (95% CI)	Adjusted HR - Full Model (95% CI)	Adjusted HR - Final Model (95% CI)
Age*			
	**0.98 (0.98–0.98), p<0.001**	0.99 (0.98–1.00), p=0.143	**0.98 (0.98–0.99), p<0.001**
Sex			
Female	Ref	Ref	-
Male	*1.11 (1.04–1.18), p=0.002*	*0.94 (0.88–1.00), p=0.052*	-
Ethnicity			
White	Ref	Ref	-
BAME	**1.16 (1.08–1.24), p<0.001**	*1.10 (1.02–1.18), p=0.010*	-
Other	1.08 (0.97–1.21), p=0.145	1.05 (0.94–1.17), p=0.405	-
Patient in employment or education			
No	Ref	Ref	Ref
Yes	*0.94 (0.88–1.00), p=0.036*	*0.93 (0.87–0.99), p=0.006*	*0.93 (0.88–1.00), p=0.038*
Psychiatric admission prior to EIP involvement			
No	Ref	Ref	Ref
Yes	**2.12 (1.98–2.26), p<0.001**	**1.97 (1.82–2.13), p<0.001**	**1.97 (1.84–2.11), p<0.001**
Average care coordinator caseload at treating EIP service*			
	**1.02 (1.01–1.02), p<0.001**	**1.02 (1.01–1.02), p<0.001**	**1.03 (1.02–1.03), p<0.001**
Likelihood that treatment began in <2 weeks (based on proportion meeting waiting time standard at treating service)*			
	1.00 (1.00–1.00), p=0.024	*1.00 (0.99–1.00), p=0.036*	-
Received Cognitive Behavioural Therapy for Psychosis			
No	Ref	Ref	-
Refused	1.04 (0.95–1.14), p=0.379	0.99 (0.90–1.09), p=0.824	-
Yes	0.99 (0.91–1.07), p=0.788	0.96 (0.92–1.09), p=0.988	-
Received Family Intervention			
No	Ref	Ref	-
Refused	*1.08 (1.00–1.16), p=0.040*	0.98 (0.89–1.05), p=0.609	-
Yes	**1.31 (1.21–1.41), p<0.001**	*1.06 (0.96–1.13), p=0.026*	-
Received carer-focussed intervention			
Not eligible	Ref	Ref	Ref
No	*1.14 (1.05–1.24), p=0.002*	1.07 (0.99–1.17), p=0.097	1.07 (0.98–1.16), p=0.109
Yes	**1.32 (1.22–1.43), p<0.001**	*1.13 (1.01–1.20), p=0.003*	**1.16 (1.07–1.25), p<0.001**
Received vocational support			
No	Ref	Ref	-
Refused	**1.14 (1.05–1.23), p=0.001**	0.98 (0.92–1.05), p=0.285	-
Yes	**1.28 (1.19–1.37), p<0.001**	*1.06 (0.99–1.15), p=0.026*	-
Received antipsychotic			
No	Ref	Ref	Ref
Yes	**1.59 (1.38–1.84), p<0.001**	*1.47 (1.27–2.52), p=0.024*	*1.26 (1.09–1.46), p=0.002*
Received clozapine			
Not eligible	Ref	Ref	Ref
Not offered	**1.64 (1.47–1.83), p<0.001**	**1.45 (1.31–1.62), p<0.001**	**1.46 (1.30–1.63), p<0.001**
Refused	**1.51 (1.25–1.83), p<0.001**	1.32 (0.97–1.47), p=0.101	1.18 (0.97–1.43), p=0.092
Yes	1.12 (0.96–1.30), p=0.138	*0.85 (0.73–0.99), p=0.042*	*0.85 (0.73–0.99), p=0.033*
Received intervention for alcohol cessation			
Not required	Ref	Ref	-
No	**0.89 (0.79–0.99), p=0.035**	0.96 (0.84–1.10), p=0.307	-
Refused	1.04 (0.93–1.18), p=0.480	0.90 (0.79–1.05), p=0.193	-

Yes	**1.40 (1.24–1.57), p<0.001**	**1.25 (1.11–1.41), p<0.001**	-
Received intervention for smoking cessation			
Not required	Ref	Ref	-
No	0.93 (0.83–1.04), p=0.187	0.92 (0.78–1.08), p=0.315	-
Refused	**1.15 (1.06–1.26), p=0.001**	0.99 (0.89–1.10), p=0.824	-
Yes	**1.24 (1.15–1.33), p<0.001**	1.02 (0.94–1.11), p=0.556	-
Received intervention for substance use			
Not required	Ref	Ref	Ref
No	*0.87 (0.77–0.99), p=0.031*	0.89 (0.72–1.09), p=0.259	0.92 (0.81–1.04), p=0.186
Refused	**1.37 (1.24–1.51), p<0.001**	**1.43 (1.25–1.63), p<0.001**	**1.34 (1.21–1.48), p<0.001**
Yes	**1.64 (1.52–1.77), p<0.001**	**1.42 (1.30–1.55), p<0.001**	**1.47 (1.36–1.59), p<0.001**
Received intervention for weight loss			
Not required	Ref	Ref	-
No	1.03 (0.87–1.22), p=0.741	1.08 (0.91–1.28), p=0.379	-
Refused	*0.79 (0.65–0.96), p=0.016*	*0.83 (0.68–1.00), p=0.053*	-
Yes	*0.92 (0.86–0.98), p=0.013*	*0.91 (0.85–0.97), p=0.006*	-
AGE x EMPLOYMENT			
AGE : EMPLOYMENT No	NA	Ref	-
AGE : EMPLOYMENT Yes	NA	*1.01 (1.00-1.01), p=0.028*	-

* For the continuous variables ‘age’, ‘care coordinator caseload’ and ‘proportion meeting waiting time standard’, stated hazard ratios indicate the change in hazard with a one-unit increase in the exposure. For example, each additional person on a care coordinator's caseload increased the hazard of CRHTT by 3% (HR 1.03, 95% CI 1.02–1.03, p<0.001).

** Interaction effects indicate the change in the hazard ratio for the second variable for each unit of change in the first. For example, for employed individuals, each additional year of age increases the hazard rate by 1% compared with those who are not employed. This suggests that the reductions in hazard rate of CRHTT referral associated with employment become less pronounced with age.

Table A7 – Detention MHA

This table presents the unadjusted and adjusted hazard ratios (HRs) with 95% confidence intervals (CIs) for the secondary outcome 'detention under the Mental Health Act'. The 'Full Model' includes all exposure variables and covariates, while the 'Final Model' is based on a refined selection of variables informed by statistical and theoretical considerations. Hazard ratios represent the relative likelihood of relapse occurring at any given time for individuals in one category of a variable compared with the reference category, holding all other variables constant. HR > 1 indicates an increased likelihood of relapse, while HR < 1 indicates a decreased likelihood. Results are adjusted for clustering within services. Results in **bold** indicate p-values ≤0.001 which were considered strong evidence. Results in *italics* indicate p-values between 0.001 and 0.05 which were considered suggestive but not definitive evidence.

Variables	Unadjusted HR (95% CI)	Adjusted HR - Full Model (95% CI)	Adjusted HR - Final Model (95% CI)
Age*			
	**0.97 (0.97–0.98), p<0.001**	1.01 (0.99–1.02), p=0.436	**0.98 (0.97–0.98), p<0.001**
Sex			
Female	Ref	Ref	-
Male	**1.23 (1.14–1.33), p<0.001**	0.96 (0.89–1.05), p=0.378	-
Ethnicity			
White	Ref	Ref	Ref
BAME	**1.63 (1.51–1.77), p<0.001**	**1.91 (1.45–2.53), p<0.001**	**1.47 (1.36–1.59), p<0.001**
Other	**1.36 (1.20–1.55), p<0.001**	*1.57 (1.05–2.65), p=0.029*	**1.27 (1.11–1.44), p<0.001**
Patient in employment or education			
No	Ref	Ref	Ref
Yes	**0.83 (0.77–0.90), p<0.001**	*0.67 (0.54–0.89), p=0.004*	**0.86 (0.80–0.93), p<0.001**
Psychiatric admission prior to EIP involvement			
No	Ref	Ref	Ref
Yes	**4.10 (3.73–4.51), p<0.001**	**3.52 (3.28–3.99), p<0.001**	**3.68 (3.34–4.05), p<0.001**
Average care coordinator caseload at treating EIP service*			
	**1.01 (1.01–1.02), p<0.001**	**1.02 (1.01–1.02), p<0.001**	**1.02 (1.01–1.02), p<0.001**
Likelihood that treatment began in <2 weeks (based on proportion meeting waiting time standard at treating service)*			
	**0.99 (0.99–1.00), p<0.001**	*1.00 (0.99–1.00), p=0.023*	-
Received Cognitive Behavioural Therapy for Psychosis			
No	Ref	Ref	Ref
Refused	*1.11 (1.00–1.23), p=0.041*	1.02 (0.91–1.14), p=0.723	1.04 (0.94–1.15), p=0.456
Yes	**0.78 (0.70–0.85), p<0.001**	**0.85 (0.74–0.91), p<0.001**	**0.85 (0.77–0.94), p=0.001**
Received Family Intervention			
No	Ref	Ref	-
Refused	*1.13 (1.04–1.23), p=0.005*	1.03 (0.94–1.13), p=0.537	-
Yes	**1.22 (1.11–1.34), p<0.001**	1.08 (0.97–1.20), p=0.138	-
Received carer-focussed intervention			
Not eligible	Ref	Ref	-
No	1.08 (0.98–1.19), p=0.123	0.98 (0.88–1.08), p=0.644	-
Yes	**1.25 (1.14–1.37), p<0.001**	1.03 (0.93–1.14), p=0.565	-
Received vocational support			
No	Ref	Ref	-
Refused	*1.16 (1.06–1.28), p=0.002*	1.00 (0.90–1.10), p=0.979	-
Yes	**1.30 (1.19–1.42), p<0.001**	1.06 (0.97–1.16), p=0.231	-
Received antipsychotic			
No	Ref	Ref	Ref
Yes	**2.09 (1.72–2.54), p<0.001**	**3.52 (1.94–6.39), p<0.001**	**1.38 (1.13–1.69), p=0.001**
Received clozapine			
Not eligible	Ref	Ref	Ref
Not offered	**1.74 (1.52–1.98), p<0.001**	**1.45 (1.27–1.65), p<0.001**	**1.45 (1.27–1.65), p<0.001**
Refused	**1.70 (1.37–2.12), p<0.001**	1.19 (0.95–1.48), p=0.128	1.20 (0.96–1.49), p=0.108
Yes	**1.23 (1.03–1.46), p<0.001**	**0.83 (0.69–0.99), p<0.001**	**0.82 (0.68–0.98), p<0.001**
Received intervention for alcohol cessation			
Not required	Ref	Ref	-
No	*0.86 (0.75–0.98), p=0.026*	0.88 (0.71–1.09), p=0.231	-
Refused	1.09 (0.94–1.26), p=0.247	0.90 (0.75–1.08), p=0.254	-

Yes	*1.17 (1.01–1.36), p=0.037*	1.02 (0.90–1.22), p=0.549	-
Received intervention for smoking cessation			
Not required	Ref	Ref	-
No	0.92 (0.80–1.05), p=0.220	0.75 (0.62–0.90), p=0.006	-
Refused	**1.24 (1.12–1.37), p<0.001**	0.99 (0.88–1.13), p=0.920	-
Yes	**1.30 (1.19–1.42), p<0.001**	1.05 (0.95–1.16), p=0.332	-
Received intervention for substance use			
Not required	Ref	Ref	Ref
No	1.01 (0.87–1.16), p=0.912	1.28 (0.76–2.15), p=0.350	1.05 (0.91–1.21), p=0.517
Refused	**1.62 (1.44–1.82), p<0.001**	**2.07 (1.34–3.19), p=0.001**	**1.55 (1.37–1.74), p<0.001**
Yes	**1.81 (1.65–1.98), p<0.001**	**1.84 (1.29–2.62), p=0.001**	**1.53 (1.39–1.67), p<0.001**
Received intervention for weight loss			
Not required	Ref	Ref	-
No	0.92 (0.75–1.14), p=0.443	0.95 (0.77–1.18), p=0.650	-
Refused	0.90 (0.73–1.12), p=0.357	0.98 (0.79–1.22), p=0.879	-
Yes	**0.81 (0.75–0.88), p<0.001**	**0.82 (0.75–0.89), p<0.001**	-
AGE x ETHNICITY			
AGE : ETHNICITY White	NA	Ref	-
AGE : ETHNICITY BAME	NA	*0.99 (0.98-0.99), p=0.042*	-
AGE : ETHNICITY Other	NA	0.99 (0.98-1.01), p=0.216	-
AGE x ANTIPSYCHOTIC			
AGE : ANTIPSYCHOTIC No	NA	Ref	-
AGE : ANTIPSYCHOTIC Yes	NA	**0.97 (0.96-0.99), p=0.001**	-

* For the continuous variables 'age', 'care coordinator caseload' and 'proportion meeting waiting time standard', stated hazard ratios indicate the change in hazard with a one-unit increase in the exposure. For example, each additional person on a care coordinator's caseload increased the hazard of detention by 2% (HR 1.02, 95% CI 1.01–1.02, p<0.001).

** Interaction effects indicate the change in the hazard ratio for the second variable for each unit of change in the first. For example, for individuals taking antipsychotic medication, each additional year of age decreases the hazard rate by 3% compared with those not taking it. This suggests that the increased hazard rate of detention associated with antipsychotic medication reduces with age.

Table A8- Mortality

This table presents the unadjusted and adjusted hazard ratios (HRs) with 95% confidence intervals (CIs) for the secondary outcome ‘mortality’. The ‘Full Model’ includes all exposure variables and covariates, while the ‘Final Model’ is based on a refined selection of variables informed by statistical and theoretical considerations. Hazard ratios represent the relative likelihood of relapse occurring at any given time for individuals in one category of a variable compared with the reference category, holding all other variables constant. HR > 1 indicates an increased likelihood of relapse, while HR < 1 indicates a decreased likelihood. Results are adjusted for clustering within services. Results in **bold** indicate p-values ≤0.001 which were considered strong evidence. Results in *italics* indicate p-values between 0.001 and 0.05 which were considered suggestive but not definitive evidence.

Variables	Unadjusted HR (95% CI)	Adjusted HR - Full Model (95% CI)	Adjusted HR - Final Model (95% CI)
Age*			
	**1.06 (1.05–1.07), p<0.001**	**1.07 (1.05–1.08), p<0.001**	**1.07 (1.05–1.08), p<0.001**
Sex			
Female	Ref	Ref	-
Male	1.17 (0.88–1.57), p=0.284	*1.37 (1.01–1.86), p=0.044*	-
Ethnicity			
White	Ref	Ref	Ref
BAME	**0.39 (0.26–0.59), p<0.001**	*0.55 (0.36–0.83), p=0.005*	**0.51 (0.34–0.77), p=0.001**
Other	*0.36 (0.18–0.74), p=0.005*	0.50 (0.25–1.03), p=0.061	0.51 (0.25–1.03), p=0.061
Patient in employment or education			
No	Ref	Ref	Ref
Yes	**0.47 (0.34–0.65), p<0.001**	*0.66 (0.48–0.93), p=0.016*	*0.60 (0.43–0.83), p=0.002*
Psychiatric admission prior to EIP involvement			
No	Ref	Ref	Ref
Yes	1.25 (0.94–1.66), p=0.124	*1.41 (1.05–1.89), p=0.021*	*1.42 (1.07–1.89), p=0.015*
Average care coordinator caseload at treating EIP service*			
	1.03 (1.00–1.06), p=0.064	1.03 (1.00–1.06), p=0.056	-
Likelihood that treatment began in <2 weeks (based on proportion meeting waiting time standard at treating service)*			
	1.01 (1.00–1.02), p=0.087	1.00 (0.99–1.01), p=0.733	-
Received Cognitive Behavioural Therapy for Psychosis			
No	Ref	Ref	-
Refused	1.12 (0.77–1.62), p=0.557	1.10 (0.74–1.63), p=0.637	-
Yes	**0.63 (0.44–0.91), p=0.013**	0.78 (0.53–1.14), p=0.201	-
Received Family Intervention			
No	Ref	Ref	-
Refused	0.88 (0.65–1.19), p=0.404	0.96 (0.69–1.33), p=0.796	-
Yes	**0.46 (0.30–0.70), p<0.001**	0.78 (0.49–1.22), p=0.273	-
Received carer-focussed intervention			
Not eligible	Ref	Ref	-
No	**0.71 (0.51–0.99), p=0.041**	0.90 (0.65–1.26), p=0.536	-
Yes	**0.46 (0.33–0.65), p<0.001**	0.71 (0.49–1.02), p=0.063	-
Received vocational support			
No	Ref	Ref	-
Refused	1.01 (0.74–1.39), p=0.931	0.96 (0.68–1.34), p=0.805	-
Yes	**0.48 (0.33–0.69), p<0.001**	0.68 (0.46–1.01), p=0.053	-
Received antipsychotic			
No	Ref	Ref	-
Yes	1.06 (0.61–1.87), p=0.826	1.14 (0.64–2.02), p=0.661	-
Received clozapine			
Not eligible	Ref	Ref	-
Not offered	1.47 (0.89–2.42), p=0.129	1.42 (0.86–2.36), p=0.170	-
Refused	0.76 (0.24–2.39), p=0.643	0.83 (0.26–2.62), p=0.751	-
Yes	0.49 (0.18–1.32), p=0.158	0.72 (0.26–1.96), p=0.519	-
Received intervention for alcohol cessation			
Not required	Ref	Ref	Ref
No	1.22 (0.76–1.95), p=0.409	1.70 (0.89–3.25), p=0.106	**1.80 (1.06–3.39), p<0.001**
Refused	**2.34 (1.55–3.54), p<0.001**	**2.55 (1.43–4.53), p=0.001**	**2.59 (1.46–4.58), p=0.001**

Yes	1.51 (0.89–2.57), p=0.131	1.09 (0.63–1.88), p=0.763	1.14 (0.66–1.97), p=0.638
Received intervention for smoking cessation			
Not required	Ref	Ref	-
No	1.08 (0.64–1.82), p=0.784	1.10 (0.53–2.29), p=0.805	-
Refused	**1.85 (1.29–2.67), p=0.001**	1.25 (0.80–1.95), p=0.335	-
Yes	**1.61 (1.15–2.25), p=0.005**	1.36 (0.94–1.96), p=0.106	-
Received intervention for substance use			
Not required	Ref	Ref	Ref
No	0.82 (0.45–1.49), p=0.522	0.66 (0.27–1.62), p=0.365	0.64 (0.29–1.44), p=0.285
Refused	1.48 (0.95–2.32), p=0.083	0.82 (0.43–1.57), p=0.550	0.96 (0.52–1.80), p=0.906
Yes	**1.84 (1.32–2.56), p<0.001**	**2.01 (1.37–2.95), p<0.001**	**2.50 (1.74–3.59), p<0.001**
Received intervention for weight loss			
Not required	Ref	Ref	-
No	2.10 (1.18–3.72), p=0.012	**2.34 (1.31–4.19), p=0.004**	**2.36 (1.32–4.22), p<0.001**
Refused	**2.37 (1.36–4.13), p=0.002**	1.76 (0.99–3.08), p=0.054	**1.85 (1.05–3.24), p<0.001**
Yes	0.93 (0.68–1.27), p=0.646	0.96 (0.69–1.33), p=0.801	0.91 (0.66–1.26), p=0.581

* For the continuous variables ‘age’, ‘care coordinator caseload’ and ‘proportion meeting waiting time standard’, stated hazard ratios indicate the change in hazard with a one-unit increase in the exposure. For example, each additional year of age increased the hazard of mortality by 7% (HR 1.07, 95% CI 1.05–1.08, p<0.001).

Table A9 – ED attendance

This table presents the unadjusted and adjusted incidence rate ratios (IRRs) with 95% confidence intervals (CIs) for the count outcome ‘emergency department attendance’. The ‘Full Model’ includes all exposure variables and covariates, while the ‘Final Model’ is based on a refined selection of variables informed by statistical and theoretical considerations. Incidence rate ratios represent the relative rate of emergency department attendance for individuals in one category of a variable compared with the reference category, holding all other variables constant. IRR > 1 indicates an increased rate of attendance, while IRR < 1 indicates a decreased rate. Results are adjusted for clustering within services. Results in **bold** indicate p-values ≤0.001, considered strong evidence. Results in *italics* indicate p-values between 0.001 and 0.05, considered suggestive but not definitive evidence.

Variables	Unadjusted IR (95% CI)	Adjusted IR - Full Model (95% CI)	Adjusted IR - Final Model (95% CI)
Age*			
	**0.98 (0.98–0.98), p<0.001**	**0.98 (0.98–0.99), p<0.001**	**0.98 (0.98–0.98), p<0.001**
Sex			
Female	Ref	Ref	Ref
Male	**0.92 (0.87–0.97), p=0.001**	**0.59 (0.50–0.69), p<0.001**	**0.78 (0.74–0.83), p<0.001**
Ethnicity			
White	Ref	Ref	-
BAME	1.03 (0.97–1.09), p=0.382	**1.41 (1.17–1.70), p<0.001**	-
Other	1.00 (0.91–1.10), p=0.970	0.94 (0.69–1.28), p=0.707	-
Patient in employment or education			
No	Ref	Ref	Ref
Yes	**0.90 (0.86–0.95), p<0.001**	1.10 (0.93–1.30), p=0.251	**0.89 (0.85–0.95), p<0.001**
Psychiatric admission prior to EIP involvement			
No	Ref	Ref	Ref
Yes	**1.24 (1.17–1.30), p<0.001**	**1.15 (1.12–1.25), p<0.001**	**1.17 (1.11–1.23), p<0.001**
Average care coordinator caseload at treating EIP service*			
	0.99 (0.99–1.00), p=0.006	*0.99 (0.99–1.00), p=0.003*	-
Likelihood that treatment began in <2 weeks (based on proportion meeting waiting time standard at treating service)*			
	1.00 (1.00–1.00), p=0.444	1.00 (0.99–1.00), p=0.144	-
Received Cognitive Behavioural Therapy for Psychosis			
No	Ref	Ref	-
Refused	**0.87 (0.80–0.94), p<0.001**	**0.88 (0.81–0.95), p=0.002**	-
Yes	0.95 (0.88–1.02), p=0.127	0.93 (0.89–1.03), p=0.209	-
Received Family Intervention			
No	Ref	Ref	-
Refused	0.97 (0.92–1.03), p=0.383	1.05 (0.87–1.28), p=0.582	-
Yes	*1.06 (0.99–1.13), p=0.100*	**1.62 (1.31–1.99), p<0.001**	-
Received carer-focussed intervention			
Not eligible	Ref	Ref	Ref
No	*0.91 (0.85–0.98), p=0.008*	*0.90 (0.84–0.97), p=0.003*	*0.90 (0.84–0.96), p=0.002*
Yes	0.95 (0.89–1.02), p=0.161	*0.91 (0.85–0.97), p=0.004*	*0.90 (0.85–0.96), p=0.002*
Received vocational support			
No	Ref	Ref	-
Refused	1.01 (0.95–1.08), p=0.786	0.98 (0.92–1.05), p=0.634	-
Yes	*1.07 (1.01–1.14), p=0.023*	1.00 (0.94–1.07), p=0.882	-
Received antipsychotic			
No	Ref	Ref	-
Yes	1.09 (0.98–1.21), p=0.114	1.10 (0.99–1.22), p=0.077	-
Received clozapine			
Not eligible	Ref	Ref	Ref
Not offered	**1.44 (1.29–1.60), p<0.001**	**1.32 (1.18–1.47), p<0.001**	**1.31 (1.18–1.46), p<0.001**
Refused	0.99 (0.82–1.20), p=0.915	0.94 (0.78–1.13), p=0.407	0.99 (0.77-1.1.12), p=0.407
Yes	*0.82 (0.72–0.94), p=0.004*	**0.75 (0.65–0.85), p<0.001**	**0.75 (0.66–0.86), p<0.001**
Received intervention for alcohol cessation			
Not required	Ref	Ref	Ref
No	1.07 (0.97–1.17), p=0.168	1.08 (0.94–1.24), p=0.303	1.10 (0.96–1.26), p=0.223
Refused	*1.16 (1.04–1.29), p=0.006*	1.05 (0.92–1.21), p=0.443	1.07 (0.94–1.23), p=0.315
Yes	**1.50 (1.34–1.68), p<0.001**	**1.36 (1.22–1.53), p<0.001**	**1.37 (1.23–1.54), p<0.001**

Received intervention for smoking cessation			
Not required	Ref	Ref	Ref
No	*1.13 (1.04–1.24), p=0.007*	1.00 (0.89–1.12), p=0.871	1.02 (0.89–1.17), p=0.768
Refused	**1.23 (1.14–1.32), p<0.001**	*1.09 (0.99–1.18), p=0.050*	1.08 (0.99–1.18), p=0.099
Yes	**1.39 (1.31–1.49), p<0.001**	**1.20 (1.12–1.28), p<0.001**	**1.18 (1.10–1.26), p<0.001**
Received intervention for substance use			
Not required	Ref	Ref	Ref
No	1.09 (0.99–1.20), p=0.088	1.02 (0.87–1.19), p=0.852	1.01 (0.86–1.18), p=0.915
Refused	**1.27 (1.16–1.39), p<0.001**	*1.16 (1.03–1.31), p=0.018*	*1.13 (1.00–1.28), p=0.049*
Yes	**1.59 (1.48–1.71), p<0.001**	**1.37 (1.26–1.48), p<0.001**	**1.37 (1.26–1.48), p<0.001**
Received intervention for weight loss			
Not required	Ref	Ref	-
No	1.12 (0.97–1.30), p=0.132	1.15 (0.99–1.33), p=0.062	-
Refused	0.92 (0.79–1.08), p=0.294	0.98 (0.85–1.15), p=0.839	-
Yes	**0.91 (0.86–0.96), p=0.001**	*0.94 (0.89–0.99), p=0.045*	-
AGE x SEX			
AGE : SEX Female	NA	Ref	-
AGE : SEX Male	NA	**1.01 (1.00-1.01), p=0.001**	-
AGE x ETHNICITY			
AGE : ETHNICITY White	NA	Ref	-
AGE : ETHNICITY BAME	NA	**0.99 (0.98-0.99), p=0.001**	-
AGE : ETHNICITY Other	NA	1.00 (0.99-1.01), p=0.872	-
AGE x FAMILY_INTERVENTION			
AGE : FAMILY_INTERVENTION No	NA	Ref	-
AGE : FAMILY_INTERVENTION Refused	NA	1.00 (0.99-1.00, p=0.540)	-
AGE : FAMILY_INTERVENTION Yes	NA	**0.98 (0.98-0.99), p<0.001**	-

* For the continuous variables 'age', 'care coordinator caseload' and 'proportion meeting waiting time standard', stated incidence ratios indicate the change in incidence rate with a one-unit increase in the exposure. For example, each additional year of age reduced the incidence rate of emergency department attendance by 2% (IR 0.98, 95% CI 0.98–0.98, p<0.001).

** Interaction effects indicate the change in the incidence rate ratio for the second variable for each unit of change in the first. For example, for male individuals, each additional year of age increased the incidence rate by 1% compared with female individuals. This suggests that the increased relative incidence rate of emergency department attendance associated with being female reduces with age.

Table A10 – General Admissions

This table presents the unadjusted and adjusted incidence rate ratios (IRRs) with 95% confidence intervals (CIs) for the count outcome ‘general hospital admission’. The ‘Full Model’ includes all exposure variables and covariates, while the ‘Final Model’ is based on a refined selection of variables informed by statistical and theoretical considerations. Incidence rate ratios represent the relative rate of emergency department attendance for individuals in one category of a variable compared with the reference category, holding all other variables constant. IRR > 1 indicates an increased rate of attendance, while IRR < 1 indicates a decreased rate. Results are adjusted for clustering within services. Results in **bold** indicate p-values ≤0.001, considered strong evidence. Results in *italics* indicate p-values between 0.001 and 0.05, considered suggestive but not definitive evidence.

Variables	Unadjusted IR (95% CI)	Adjusted IR - Full Model (95% CI)	Adjusted IR - Final Model (95% CI)
Age*			
	1.01 (1.01–1.01), p=0.019	1.00 (0.99–1.00), p=0.474	-
Sex			
Female	Ref	Ref	-
Male	**0.74 (0.69–0.79), p<0.001**	**0.49 (0.40–0.59), p<0.001**	**0.67 (0.62–0.71), p<0.001**
Ethnicity			
White	Ref	Ref	Ref
BAME	**0.85 (0.79–0.91), p<0.001**	**1.35 (1.08–1.69), p=0.010**	**0.85 (0.79–0.91), p<0.001**
Other	*0.87 (0.78–0.97), p=0.016*	0.74 (0.52–1.07), p=0.109	0.89 (0.80–1.00), p=0.053
Patient in employment or education			
No	Ref	Ref	Ref
Yes	**0.82 (0.77–0.87), p<0.001**	**0.80 (0.79–0.90), p<0.001**	**0.84 (0.79–0.89), p<0.001**
Psychiatric admission prior to EIP involvement			
No	Ref	Ref	Ref
Yes	**1.32 (1.24–1.41), p<0.001**	**1.32 (1.24–1.41), p<0.001**	**1.34 (1.26–1.43), p<0.001**
Average care coordinator caseload at treating EIP service*			
	1.01 (1.00–1.01), p=0.018	1.00 (1.00–1.01), p=0.093	-
Likelihood that treatment began in <2 weeks (based on proportion meeting waiting time standard at treating service)*			
	**1.01 (1.01–1.01), p<0.001**	**1.01 (1.00–1.01), p<0.001**	-
Received Cognitive Behavioural Therapy for Psychosis			
No	Ref	Ref	-
Refused	0.94 (0.86–1.03), p=0.191	0.92 (0.83–1.01), p=0.076	-
Yes	*0.91 (0.84–0.99), p=0.031*	*0.90 (0.82–0.98), p=0.014*	-
Received Family Intervention			
No	Ref	Ref	-
Refused	0.99 (0.92–1.07), p=0.870	0.96 (0.89–1.03), p=0.264	-
Yes	0.98 (0.90–1.06), p=0.612	0.96 (0.88–1.05), p=0.319	-
Received carer-focussed intervention			
Not eligible	Ref	Ref	-
No	1.00 (0.93–1.09), p=0.910	1.02 (0.94–1.11), p=0.622	-
Yes	1.02 (0.94–1.10), p=0.684	1.04 (0.96–1.13), p=0.324	-
Received vocational support			
No	Ref	Ref	-
Refused	1.02 (0.95–1.11), p=0.548	1.00 (0.93–1.09), p=0.904	-
Yes	0.95 (0.88–1.02), p=0.136	0.95 (0.88–1.03), p=0.183	-
Received antipsychotic			
No	Ref	Ref	-
Yes	1.13 (1.00–1.28), p=0.052	1.10 (0.97–1.25), p=0.123	-
Received clozapine			
Not eligible	Ref	Ref	-
Not offered	**1.33 (1.17–1.51), p<0.001**	**1.26 (1.11–1.43), p<0.001**	-
Refused	1.00 (0.80–1.26), p=0.998	1.03 (0.83–1.29), p=0.809	-
Yes	1.08 (0.93–1.27), p=0.331	1.03 (0.88–1.21), p=0.712	-
Received intervention for alcohol cessation			
Not required	Ref	Ref	Ref
No	0.91 (0.82–1.02), p=0.093	1.04 (0.87–1.23), p=0.692	1.03 (0.87–1.22), p=0.765
Refused	1.10 (0.97–1.25), p=0.128	1.02 (0.87–1.21), p=0.785	1.03 (0.88–1.22), p=0.706
Yes	**1.42 (1.24–1.62), p<0.001**	**1.26 (1.10–1.44), p=0.001**	**1.29 (1.13–1.47), p<0.001**

Received intervention for smoking cessation			
Not required	Ref	Ref	Ref
No	0.90 (0.81–1.00), p=0.051	0.88 (0.75–1.04), p=0.133	0.87 (0.74–1.03), p=0.104
Refused	*1.12 (1.03–1.23), p=0.012*	1.00 (0.91–1.12), p=0.927	1.01 (0.91–1.12), p=0.899
Yes	**1.32 (1.23–1.43), p<0.001**	**1.21 (1.12–1.31), p<0.001**	**1.22 (1.12–1.32), p<0.001**
Received intervention for substance use			
Not required	Ref	Ref	Ref
No	*0.88 (0.79–0.99), p=0.033*	1.05 (0.87–1.28), p=0.617	1.04 (0.86–1.26), p=0.720
Refused	*1.12 (1.01–1.25), p=0.037*	*1.20 (1.03–1.39), p=0.017*	1.16 (1.00–1.34), p=0.053
Yes	**1.27 (1.16–1.38), p<0.001**	**1.27 (1.16–1.40), p<0.001**	**1.24 (1.13–1.36), p<0.001**
Received intervention for weight loss			
Not required	Ref	Ref	-
No	1.15 (0.97–1.38), p=0.112	1.16 (0.98–1.38), p=0.086	-
Refused	0.98 (0.82–1.18), p=0.852	0.93 (0.78–1.12), p=0.437	-
Yes	*1.08 (1.01–1.15), p=0.028*	1.04 (0.97–1.12), p=0.242	-
AGE x SEX			
AGE : SEX Female	NA	Ref	-
AGE : SEX Male	NA	*1.01 (1.00-1.01), p=0.002*	-
AGE x ETHNICITY			
AGE : ETHNICITY White	NA	Ref	-
AGE : ETHNICITY BAME	NA	**0.99 (0.98-0.99), p=<0.001**	-
AGE : ETHNICITY Other	NA	1.00 (0.99-1.01), p=0.872	-

* For the continuous variables 'age', 'care coordinator caseload' and 'proportion meeting waiting time standard', stated incidence ratios indicate the change in incidence rate with a one-unit increase in the exposure. However, none of these variables were significantly associated with rates of general hospital admission in the final model.

** Interaction effects indicate the change in the incidence rate ratio for the second variable for each unit of change in the first. For example, for male individuals, each additional year of age increased the incidence rate by 1% compared with female individuals. This suggests that the increased relative incidence rate of general hospital admission associated with being female reduces with age.

MISSING DATA

Our chosen outcomes are mandatory submissions for NHS England. The absence of data for an outcome was interpreted by NHS England as the patient not having experienced the outcome. There is a possibility that in some cases, missing data was due to incomplete or incorrect recording rather than a true absence of the outcome. If the likelihood of recording was associated with patient characteristics or outcomes, this could introduce a risk of informative censoring. However, due to the nature of the data, it was not possible to test for or fully address this potential bias.

The data supplied from the NCAP were extremely comprehensive with very few missing values – the only exceptions were the service-level exposure variables (care coordinator caseload per service and proportion meeting waiting time standard per service) which had been compiled from sources other than the case-note audit. For these two variables, we compared those with available data and those with missing data, on the other variables with available data – including demographics, exposures and outcomes. Data were compared visually in missing data matrices and using statistical tests. Proportions and patterns of missing data for all variables, and the results of these comparisons are displayed in the appendix.

The profile of missingness indicated that data for the service-level exposure variables were likely to be Missing At Random (i.e. missingness related to service characteristics) rather than Missing Completely At Random – using complete cases only for subsequent analyses would therefore risk proportionally dropping more participants from specific services and compromise our conclusions if specific participant characteristics (and associated outcomes) were overrepresented at these services. Therefore, for these variables we applied multiple imputation using chained equations ('mice' package in R), leveraging the observed data to generate plausible values for missing data. We created 20 imputed datasets and pooled results according to Rubin's rules. We then repeated all final analyses substituting the maximum and minimum observed values for missing values for these variables to generate 'worst-case' and 'best-case' scenarios and assess the effect on the conclusions from our analyses. Further details of the multiple imputation procedure and results from the sensitivity analyses are reported below.

Therefore, note that although there appear to be large amounts of missing data for time-to-event outcomes in the figures below (TIME_TO_RELAPSE, TIME_TO_REFERRAL_CRHTT, TIME_TO_ADMISSION_PSYCH), this was presumed due to patients not having experienced the events in question (i.e. they were missing if the patient had not been admitted or referred to CRHTT in the follow-up period).

Figure A5 – Missing Data Proportions

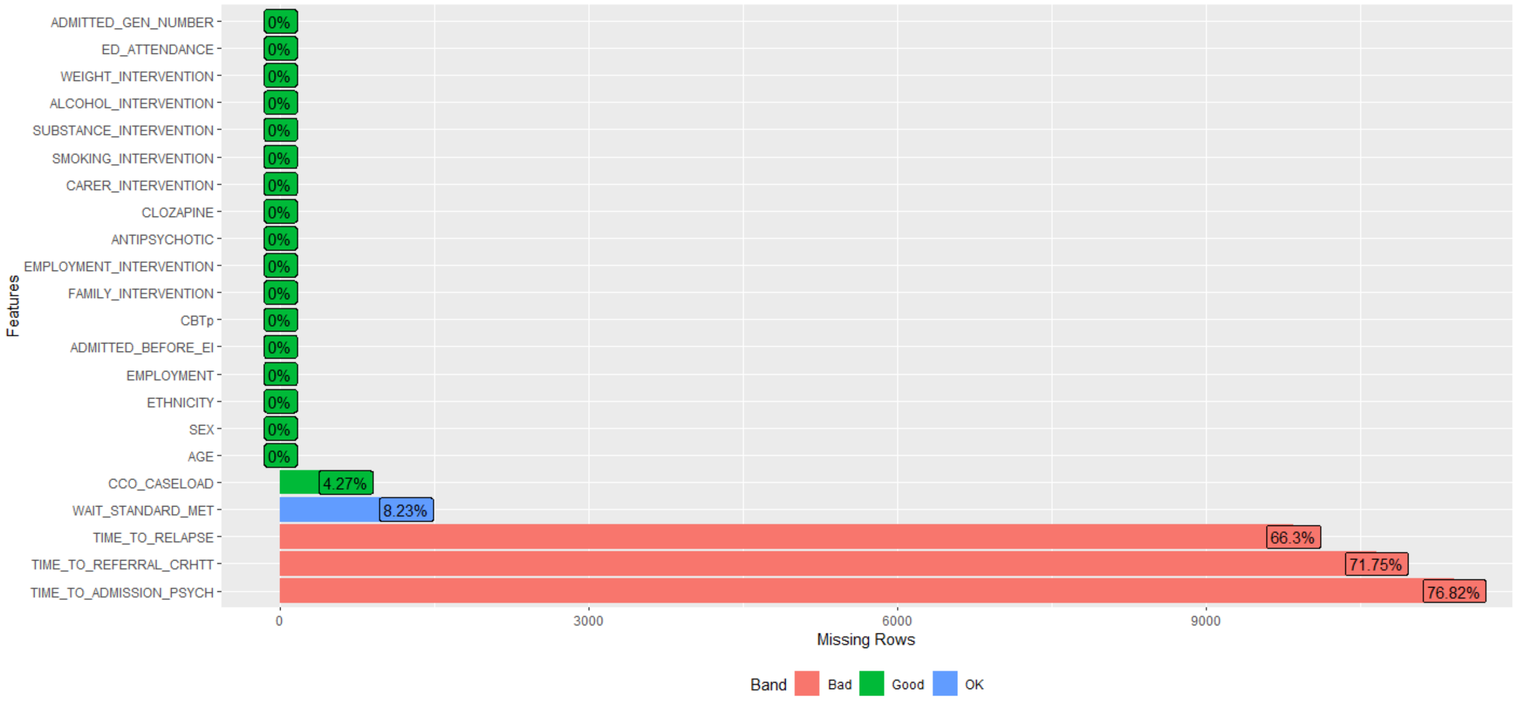

Figure A6 – Missing Value Map

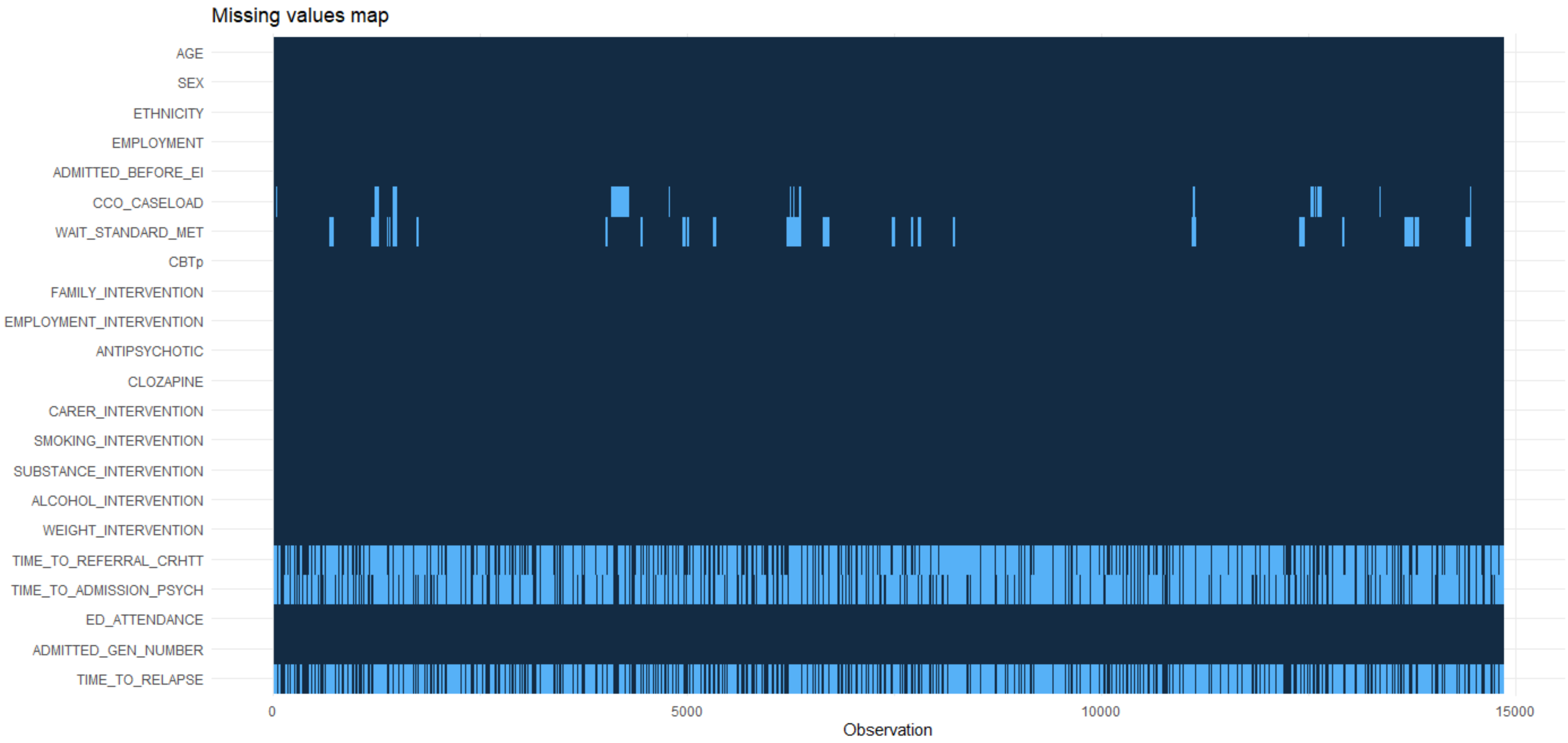

Details of Multiple Imputation Procedure

1. **Software and Rationale**:
   - The multiple imputation was conducted using R with the 'mice' package. Twenty imputed datasets were generated based on the assumption that missing data were missing at random (MAR).
2. **Imputation Model**:
   - The imputation model included the following variables: demographic characteristics (age, sex, ethnicity, employment status), clinical severity indicators (inpatient admission before EIP), and service-level variables (care coordinator caseload size, proportion meeting waiting time standard).
   - Predictive mean matching was used for continuous variables.
3. **Convergence and Diagnostics**:
   - Each dataset was imputed over 50 iterations, and diagnostic checks (trace plots) confirmed stable parameter estimates. Convergence was assessed visually.
4. **Transformations**:
   - Continuous variables were log-transformed prior to imputation to correct skewness. Imputed values were back-transformed for analysis.
5. **Post-Imputation Analysis**:
   - Rubin's rules were used to pool parameter estimates and standard errors across the 20 imputed datasets. Sensitivity analyses using complete case analysis and mean imputation confirmed consistency with the primary results.
6. **Missing Data Summary**:
   - A total of 4.27% of observations for care coordinator caseload size were missing. A total of 8.23% of observations for proportion meeting waiting time standard were missing. The distribution of imputed values was consistent with the observed data.
7. **Imputation Diagnostics**:
   - Density plots and summary statistics were used to compare observed and imputed values for care coordinator caseload size and waiting time standard. Unfortunately these were not cleared for extraction from the ONS secure research service environment due to containing potentially identifiable data.

SENSITIVITY ANALYSES

We conducted the following sensitivity analyses for this study:

Sensitivity analysis 1: Imputing the maximum observed value in place of missing values for the continuous service-level variable ‘average care coordinator caseload’.

Sensitivity analysis 2: Imputing the minimum observed value in place of missing values for the continuous service-level variable ‘average care coordinator caseload’.

The results of these sensitivity analyses are displayed in the tables below (primary outcome only). None of the conclusions of our study were altered by this analysis. The magnitude of the effect sizes for the association with relapse differed slightly when minimum values were imputed.

The tables below present the unadjusted and adjusted hazard ratios (HRs) with 95% confidence intervals (CIs) for the primary outcome (relapse, defined as inpatient admission or CRHTT referral). The ‘Full Model’ includes all exposure variables and covariates, while the ‘Final Model’ is based on a refined selection of variables informed by statistical and theoretical considerations. Hazard ratios represent the relative likelihood of relapse occurring at any given time for individuals in one category of a variable compared with the reference category, holding all other variables constant. HR > 1 indicates an increased likelihood of relapse, while HR < 1 indicates a decreased likelihood. Results are adjusted for clustering within services. Results in **bold** indicate p-values ≤0.001 which were considered strong evidence. Results in *italics* indicate p-values between 0.001 and 0.05 which were considered suggestive but not definitive evidence.

Care coordinator caseload – maximum value imputed

Variables	Unadjusted HR (95% CI)	Adjusted HR - Full Model (95% CI)	Adjusted HR - Final Model (95% CI)
Age*			
	**0.98 (0.98–0.98), p<0.001**	*0.98 (0.96–1.00), p=0.034*	**0.98 (0.98–0.99), p<0.001**
Sex			
Female	Ref	NA	-
Male	**1.15 (1.08–1.21), p<0.001**	0.95 (0.89–1.00), p=0.071	-
Ethnicity			
White	Ref	NA	NA
BAME	**1.25 (1.18–1.33), p<0.001**	**1.17 (1.11–1.24), p<0.001**	**1.19 (1.12–1.26), p<0.001**
Other	*1.13 (1.02–1.24), p=0.020*	1.07 (0.97–1.19), p=0.176	1.08 (0.98–1.19), p=0.128
Patient in employment or education			
No	Ref	NA	NA
Yes	**0.90 (0.85–0.96), p=0.001**	**0.73 (0.61–0.88), p=0.001**	*0.89 (0.80–0.95), p=0.005*
Psychiatric admission prior to EIP involvement			
No	Ref	NA	NA
Yes	**2.43 (2.29–2.58), p<0.001**	**2.22 (2.09–2.36), p<0.001**	**2.26 (2.12–2.41), p<0.001**
Average care coordinator caseload at treating EIP service*			
	**1.02 (1.01–1.02), p<0.001**	0.99 (0.99–1.00), p=0.080	**1.02 (1.01–1.02), p<0.001**
Likelihood that treatment began in <2 weeks (based on proportion meeting waiting time standard at treating service)*			
	1.00 (1.00–1.00), p=0.05	1.00 (1.00–1.00), p=0.118	-
Received Cognitive Behavioural Therapy for Psychosis			
No	Ref	NA	-
Refused	1.05 (0.97–1.14), p=0.202	1.00 (0.92–1.09), p=0.958	-
Yes	*0.92 (0.85–0.99), p=0.021*	0.95 (0.88–1.02), p=0.178	-
Received Family Intervention			
No	Ref	NA	-

Refused	*1.07 (1.01–1.15), p=0.031*	0.99 (0.92–1.07), p=0.833	-
Yes	**1.24 (1.15–1.33), p<0.001**	1.07 (0.97–1.17), p=0.082	-
Received carer-focussed intervention			
Not eligible	Ref	NA	-
No	*1.11 (1.03–1.20), p=0.006*	0.99 (0.91–1.08), p=0.355	-
Yes	**1.28 (1.19–1.37), p<0.001**	*1.09 (1.01–1.18), p=0.031*	-
Received vocational support			
No	Ref	NA	-
Refused	**1.12 (1.07–1.18), p<0.001**	1.01 (0.92–1.11), p=0.120	-
Yes	**1.23 (1.18–1.29), p<0.001**	*1.09 (1.02–1.17), p=0.015*	-
Received antipsychotic			
No	Ref	NA	NA
Yes	**2.20 (2.00–2.41), p<0.001**	**2.36 (2.12–2.62), p<0.001**	**1.33 (1.16–1.52), p<0.001**
Received clozapine			
Not eligible	Ref	NA	NA
Not offered	**1.36 (1.25–1.47), p<0.001**	**1.51 (1.36–1.67), p<0.001**	**1.52 (1.37–1.68), p<0.001**
Refused	**1.63 (1.43–1.85), p<0.001**	**1.36 (1.15–1.61), p<0.001**	**1.40 (1.18–1.65), p<0.001**
Yes	**1.74 (1.60–1.90), p<0.001**	0.94 (0.84–1.10), p=0.631	0.98 (0.86–1.12), p=0.766
Received intervention for alcohol cessation			
Not required	Ref	NA	-
No	**0.85 (0.79–0.91), p<0.001**	1.00 (0.85–1.11), p=0.989	-
Refused	**0.82 (0.75–0.89), p<0.001**	0.89 (0.78–1.04), p=0.156	-
Yes	1.06 (0.98–1.15), p=0.142	**1.19 (1.06–1.33), p=0.003**	-
Received intervention for smoking cessation			
Not required	Ref	NA	-
No	**0.89 (0.83–0.96), p<0.001**	0.94 (0.81–1.09), p=0.373	-
Refused	1.00 (0.95–1.06), p=0.996	1.04 (0.92–1.11), p=0.310	-
Yes	**1.09 (1.04–1.14), p<0.001**	*1.19 (1.09–1.28), p=0.003*	-
Received intervention for substance use			
Not required	Ref	NA	NA
No	**0.85 (0.79–0.92), p<0.001**	1.02 (0.92–1.14), p=0.730	1.02 (0.91–1.13), p=0.775
Refused	1.01 (0.95–1.09), p=0.697	**1.47 (1.34–1.61), p<0.001**	**1.53 (1.43–1.65), p<0.001**
Yes	**1.17 (1.11–1.23), p<0.001**	**1.46 (1.35–1.58), p<0.001**	**1.54 (1.43–1.65), p<0.001**
Received intervention for weight loss			
Not required	Ref	NA	NA
No	1.02 (0.92–1.14), p=0.695	0.98 (0.92–1.06), p=0.355	-
Refused	1.01 (0.91–1.13), p=0.875	0.91 (0.77–1.07), p=0.254	-
Yes	**1.15 (1.10–1.19), p<0.001**	**0.84 (0.79–0.89), p<0.001**	-
AGE x EMPLOYMENT			
AGE : EMPLOYMENT No	NA	NA	-
AGE : EMPLOYMENT Yes	NA	*1.01 (1.00-1.01), p=0.010*	-
AGE x ANTIPSYCHOTIC			
AGE : ANTIPSYCHOTIC No	NA	NA	-
AGE : ANTIPSYCHOTIC Yes	NA	*0.98 (0.97-0.99), p=0.002*	-

* For the continuous variables ‘age’, ‘care coordinator caseload’ and ‘proportion meeting waiting time standard’, stated hazard ratios indicate the change in hazard with a one-unit increase in the exposure. For example, each additional person on a care coordinator's caseload increased the hazard of relapse by 2% (HR 1.02, 95% CI 1.01–1.02, p<0.001).

** Interaction effects indicate the change in the hazard ratio for the second variable for each unit of change in the first. For example, for individuals taking antipsychotic medication, each additional year of age decreases the hazard rate by 2% compared with those not taking it. This suggests that the increased hazard rate of relapse associated with antipsychotic medication reduces with age.

Care coordinator caseload – minimum value imputed

Variables	Unadjusted HR (95% CI)	Adjusted HR - Full Model (95% CI)	Adjusted HR - Final Model (95% CI)
Age*			
	**0.98 (0.98–0.98), p<0.001**	1.00 (0.99–1.02), p=0.495	**0.98 (0.98–0.98), p<0.001**
Sex			
Female	Ref	NA	-
Male	**1.15 (1.08–1.21), p<0.001**	0.95 (0.89–1.01), p=0.073	-
Ethnicity			
White	Ref	NA	NA
BAME	**1.25 (1.18–1.33), p<0.001**	**1.16 (1.08–1.23), p<0.001**	**1.13 (1.06–1.21), p<0.001**
Other	*1.13 (1.02–1.24), p=0.020*	1.07 (0.97–1.18), p=0.190	1.07 (0.96–1.17), p=0.247
Patient in employment or education			
No	Ref	NA	NA
Yes	**0.90 (0.85–0.96), p=0.001**	**0.73 (0.61–0.88), p=0.001**	*0.92 (0.87–0.97), p=0.006*
Psychiatric admission prior to EIP involvement			
No	Ref	NA	NA
Yes	**2.43 (2.29–2.58), p<0.001**	**2.22 (2.08–2.37), p<0.001**	**2.28 (2.14–2.43), p<0.001**
Average care coordinator caseload at treating EIP service*			
	**1.02 (1.01–1.02), p<0.001**	**1.02 (1.01–1.02), p<0.001**	**1.03 (1.02–1.03), p<0.001**
Likelihood that treatment began in <2 weeks (based on proportion meeting waiting time standard at treating service)*			
	1.00 (1.00–1.00), p=0.05	1.00 (1.00–1.00), p=0.560	-
Received Cognitive Behavioural Therapy for Psychosis			
No	Ref	NA	-
Refused	1.05 (0.97–1.14), p=0.202	1.00 (0.91–1.09), p=0.925	-
Yes	*0.92 (0.85–0.99), p=0.021*	0.95 (0.88–1.02), p=0.171	-
Received Family Intervention			
No	Ref	NA	-
Refused	*1.07 (1.01–1.15), p=0.031*	1.00 (0.93–1.07), p=0.910	-
Yes	**1.24 (1.15–1.33), p<0.001**	1.07 (0.99–1.16), p=0.070	-
Received carer-focussed intervention			
Not eligible	Ref	NA	-
No	*1.11 (1.03–1.20), p=0.006*	1.03 (0.96–1.11), p=0.408	-
Yes	**1.28 (1.19–1.37), p<0.001**	*1.09 (1.01–1.17), p=0.028*	-
Received vocational support			
No	Ref	NA	-
Refused	**1.12 (1.07–1.18), p<0.001**	1.06 (0.98–1.14), p=0.125	-
Yes	**1.23 (1.18–1.29), p<0.001**	*1.09 (1.02–1.16), p=0.016*	-
Received antipsychotic			
No	Ref	NA	NA
Yes	**2.20 (2.00–2.41), p<0.001**	**2.36 (1.58–3.51), p<0.001**	**1.33 (1.16–1.52), p<0.001**
Received clozapine			
Not eligible	Ref	NA	NA
Not offered	**1.36 (1.25–1.47), p<0.001**	**1.50 (1.36–1.66), p<0.001**	**1.48 (1.33–1.63), p<0.001**
Refused	**1.63 (1.43–1.85), p<0.001**	**1.36 (1.15–1.61), p<0.001**	**1.40 (1.18–1.65), p<0.001**
Yes	**1.74 (1.60–1.90), p<0.001**	0.97 (0.85–1.11), p=0.626	0.97 (0.85–1.11), p=0.643
Received intervention for alcohol cessation			
Not required	Ref	NA	NA
No	**0.85 (0.79–0.91), p<0.001**	1.00 (0.85–1.11), p=0.980	-
Refused	**0.82 (0.75–0.89), p<0.001**	0.90 (0.78–1.04), p=0.154	-
Yes	1.06 (0.98–1.15), p=0.142	**1.19 (1.06–1.33), p=0.003**	-
Received intervention for smoking cessation			
Not required	Ref	NA	NA
No	**0.89 (0.83–0.96), p<0.001**	0.93 (0.80–1.08), p=0.367	-
Refused	1.00 (0.95–1.06), p=0.996	1.01 (0.92–1.11), p=0.847	-
Yes	**1.09 (1.04–1.14), p<0.001**	1.04 (0.96–1.12), p=0.316	-
Received intervention for substance use			
Not required	Ref	NA	NA
No	**0.85 (0.79–0.92), p<0.001**	1.03 (0.86–1.24), p=0.724	0.99 (0.89–1.10), p=0.862
Refused	1.01 (0.95–1.09), p=0.697	**1.49 (1.31–1.68), p<0.001**	**1.43 (1.30–1.57), p<0.001**
Yes	**1.17 (1.11–1.23), p<0.001**	**1.46 (1.35–1.58), p<0.001**	**1.50 (1.40–1.62), p<0.001**
Received intervention for weight loss			
Not required	Ref	NA	NA
No	1.02 (0.92–1.14), p=0.695	1.07 (0.92–1.25), p=0.385	-

Refused	1.01 (0.91–1.13), p=0.875	0.91 (0.77–1.07), p=0.266	-
Yes	**1.15 (1.10–1.19), p<0.001**	**0.89 (0.84–0.95), p=0.001**	-
AGE x EMPLOYMENT			
AGE : EMPLOYMENT No	NA	NA	-
AGE : EMPLOYMENT Yes	NA	*1.01 (1.00-1.01), p=0.011*	-
AGE x ANTIPSYCHOTIC			
AGE : ANTIPSYCHOTIC No	NA	NA	-
AGE : ANTIPSYCHOTIC Yes	NA	**0.98 (0.97-0.99), p=0.001**	-

* For the continuous variables ‘age’, ‘care coordinator caseload’ and ‘proportion meeting waiting time standard’, stated hazard ratios indicate the change in hazard with a one-unit increase in the exposure. For example, each additional person on a care coordinator's caseload increased the hazard of relapse by 3% (HR 1.03, 95% CI 1.02–1.03, p<0.001).

** Interaction effects indicate the change in the hazard ratio for the second variable for each unit of change in the first. For example, for individuals taking antipsychotic medication, each additional year of age decreases the hazard rate by 2% compared with those not taking it. This suggests that the increased hazard rate of relapse associated with antipsychotic medication reduces with age.

REFERENCES (appendix)

1. Williams R, Penington E, Gupta V, Tsiachristas A, French P, Lennox B, et al. Predictors of positive outcomes from 'Early Intervention in Psychosis': protocol for a national retrospective cohort study. *Front Psychiatry* 2023; **14**: 1274820.

2. von Elm E, Altman DG, Egger M, Pocock SJ, Gotzsche PC, Vandenbroucke JP, et al. The Strengthening the Reporting of Observational Studies in Epidemiology (STROBE) statement: guidelines for reporting observational studies. *Lancet* 2007; **370**(9596): 1453-7.

3. R Core Team. R: A Language and Environment for Statistical Computing. *R Foundation for Statistical Computing, Vienna, Austria*.

4. Tennant PWG, Murray EJ, Arnold KF, Berrie L, Fox MP, Gadd SC, et al. Use of directed acyclic graphs (DAGs) to identify confounders in applied health research: review and recommendations. *Int J Epidemiol* 2021; **50**(2): 620-32.

5. Burnham K, Anderson D. Model-selection uncertainty with examples. Model selection and multimodel inference: a practical information-theoretic approach. New York, NY: Springer New York; 1998.

6. Johnson S, Lamb D, Marston L, Osborn D, Mason O, Henderson C, et al. Peer-supported self-management for people discharged from a mental health crisis team: a randomised controlled trial. *Lancet* 2018; **392**(10145): 409-18.

7. Hett D, Morales-Munoz I, Durdurak BB, Carlish M, Marwaha S. Rates and associations of relapse over 5 years of 2649 people with bipolar disorder: a retrospective UK cohort study. *Int J Bipolar Disord* 2023; **11**(1): 23.

8. Puntis S, Oke J, Lennox B. Discharge pathways and relapse following treatment from early intervention in psychosis services. *BJPsych Open* 2018; **4**(5): 368-74.

9. Colquhoun D. An investigation of the false discovery rate and the misinterpretation of p-values. *R Soc Open Sci* 2014; **1**(3): 140216.

10. Austin PC, Steyerberg EW. Events per variable (EPV) and the relative performance of different strategies for estimating the out-of-sample validity of logistic regression models. *Stat Methods Med Res* 2017; **26**(2): 796-808.
